# DNA fingerprinting, fixation-index (Fst), and admixture mapping of selected Bambara groundnut (*Vigna subterranea* [L.] Verdc.) accessions using ISSR markers system

**DOI:** 10.1038/s41598-021-93867-5

**Published:** 2021-07-15

**Authors:** Md Mahmudul Hasan Khan, Mohd Y. Rafii, Shairul Izan Ramlee, Mashitah Jusoh, Md Al Mamun, Jamilu Halidu

**Affiliations:** 1 Laboratory of Climate-Smart Food Crop Production, Institute of Tropical Agriculture and Food Security (ITAFoS), Universiti Putra Malaysia (UPM), UPM Serdang, 43400 Selangor, Malaysia; 2 Department of Crop Science, Faculty of Agriculture, Universiti Putra Malaysia (UPM), UPM Serdang, 43400 Selangor, Malaysia; 3 Bangladesh Agricultural Research Institute (BARI), Gazipur, 1701 Bangladesh; 4 Bangladesh Jute Research Institute (BJRI), Dhaka, Bangladesh

**Keywords:** Plant molecular biology, Plant breeding, Plant sciences

## Abstract

As a new crop in Malaysia, forty-four Bambara groundnut (*Vigna subterranea* L. verdc.) genotypes were sampled from eleven distinct populations of different origins to explore the genetic structure, genetic inconsistency, and fixation index. The Bambara groundnut, an African underutilized legume, has the capacity to boost food and nutrition security while simultaneously addressing environmental sustainability, food availability, and economic inequalities. A set of 32 ISSRs were screened out of 96 primers based on very sharp, clear, and reproducible bands which detected a total of 510 loci with an average of 97.64% polymorphism. The average calculated value of *PIC* = 0.243, *RP* = 5.30, *H* = 0.285, and *MI* = 0.675 representing the efficiency of primer set for genetic differentiation among the genotypes. The ISSR primers revealed the number of alleles (*Na* = 1.97), the effective number of alleles (*Ne* = 1.38), Nei's genetic diversity (*h* = 0.248), and a moderate level of gene flow (*Nm* = 2.26) across the genotypes studied. The estimated Shannon’s information index (*I* = 0.395) indicates a high level of genetic variation exists among the accessions. Based on Nei’s genetic dissimilarity a UPMGA phylogenetic tree was constructed and grouped the entire genotypes into 3 major clusters and 6 subclusters. PCA analysis revealed that first principal component extracted maximum variation (PC1 = 13.92%) than second principal component (PC2 = 12.59%). Bayesian model-based STRUCTURE analysis assembled the genotypes into 3 (best ΔK = 3) genetic groups. The fixation-index (F*st*) analysis narrated a very great genetic diversity (F*st* = 0.19 to 0.40) exists within the accessions of these 3 clusters. This investigation specifies the effectiveness of the ISSR primers system for the molecular portrayal of *V. subterranea* genotypes that could be used for genetic diversity valuation, detection, and tagging of potential genotypes with quick, precise, and authentic measures for this crop improvement through effective breeding schemes.

## Introduction

Bambara groundnut (*Vigna subterranea* L. Verdc.) is a potential legume recently introduce in Malaysia from the African continent. It is the third most important legume in Africa after groundnut and cowpea^1^ popularly known as “poor man’s crop” or “women’s crop” nowadays declare as “crop for the new millennium”^2^. It is one of the most drought-tolerant legumes also well fitted to low fertile soil where other major crops cannot survive easily ^1^. Besides Africa, there is ample scope to introducing this legume in Asian countries such as Malaysia, Bangladesh, India, Thailand, and Indonesia. The major drawbacks of this crop expansion in lack of high-yielding cultivars' improved production technologies as well as limited research interest by the global scientific community^3^. The yield of Bambara ground is recorded as low as 68.5 to 159.9 kg ha^-1^^4^, 650–850 kg ha^-1^^5^ are smaller than other legumes. Although this crop has enabled to produce up to 4.0 t ha^-1^^6^ whereas Redjeki^7^ recorded 0.7–2.0 t ha^-1^ and 0.38–1.6 t ha^-1^ reported by Khan et al.^2^ at the optimal growing environment. This legume has versatile usages and the ability to supply agri-based food and nutrients. It can be a potential source of cheap protein for resources-limited consumers and growers^8^ where animal-based protein is very expensive. Seeds of Bambara groundnut can be consumed in a fresh form as vegetables whereas dried seeds are processed as roasted snacks^3^. Bambara groundnut is considered as “complete food” due to the content of a significant amount of nutrients such as Carbohydrates (64.4%), Protein (23.6%), fat (6.5%), fiber (5.5%)^9^, and trace elements. Moreover, it possesses a countable amount of K (11.44–19.35 mg/100 g), Fe (4.9–48 mg/100 g), Na (2.9–12.0 mg/100 g), and Ca (95.8–99 mg/100 g), all this amount is favourably comparable to other consumable legumes ^10^. The average yield of Bambara groundnut is not remarkable yet due to the fact of the use of local landraces for cultivation. No high-yielding cultivars were released by the breeding scheme due to their very complex floral biology (cleistogamous) and anthesis period (3.00 to 5.30 am) reported by Suwanprasert et al^11^. Assessment of genetic diversity is the prerequisite for genetic enhancement of any crop’s species especially in an under-research crop like Bambara groundnut. ^5^. For effective breeding in Bambara groundnut morphological alongside molecular characterization of existing germplasm or landraces becomes authoritative. A few molecular types of research has been demonstrated for this crop improvement such as SDS–polyacrylamide electrophoresis technique by Odeigah and Osanyinpeju, ^12^, DArT used by Olukolu et al*.* ^5^, RAPD used by Rungnoi et al*.* ^1^, Amadou et al*.* ^13^, Massawe et al*.* ^14^, and Mukakalisa et al*.* ^15^, AFLP used by Massawe et al*.* ^16^ and Ntundu et al*.* ^17^, SSR used by Basu et al*.* ^18^, Somta et al*.* ^19^, Siise Aliyu and Massawe, ^20^, Molosiwa et al*.* ^21^, Odongo et al.^22^, Mohammed et al*.* ^3^ and all of these researchers noted the existence of greater diversity of Bambara groundnut species. Like other underutilized legumes, there is only one research has been conducted on Bambara groundnut using ISSR molecular marker by Rungnoi et al*.* ^1^. For the development of new cultivars information related to genetic relatedness among the prospective parents is obligatory. Before commencing breeding, program information related to the genetic distance of parents must be discover based on both morphological and molecular approaches ^23^. Genetic diversity can be assessed considering both qualitative and quantitative features ^16^ though characterization based on morphological traits is less reliable due to influenced by environmental factors ^24^. Molecular marker provides more precise and authentic results to detecting variation that exists in the landraces compared to that of conventional approaches ^25^. Moreover, molecular markers can be used for tracing the genotypes' origin as well as be employed in plant breeding programs^26^. One of the effective molecular markers is ISSR (inter simple sequence repeat) that can be successfully appley to plant genome identification and characterization. The main advantage of ISSR is it produces multiple bands in the same locus, highly reproducible, and does not need any previous genomic information of the Plant^27^. There is no information on the use of ISSR primers in Bambara groundnut genetic diversity, as well as its botany, genetics, farming techniques, economic value addition due to the very recent introduction of this legume in Malaysia. In the present study, we used ISSR primers to measure the extent of genetic variation, genetic drift as well as genetic admixture among the existing accessions. Additionally, the findings of this research will enrich the molecular database of Bambara groundnut in Malaysian content. It also provides a basic idea of conserving and protecting the existing genotypes through optimum utilization as well as an effective breeding program for *V. subterranea* improvement..

## Materials and Methods

### Plant materials

The current study was conducted at the Institute of Tropical Agriculture and Food Security (ITAFoS), Universiti Putra Malaysia (UPM), with GPS of 2°98′26.9′′N and 101°73′58.9′′E, from June to December 2020. Initially, fifteen collected accessions were undertaken the formal identification by Md Mahmudul Hasan Khan under the direct supervision of Prof. Dr. Mohd. Rafii Yusop, Director, ITAFoS, UPM, Malaysia with following the proper national and international strategies and deposited these accessions at GenBank, ITAFoS, UPM. For this study eleven accessions were used from the “ITAFoS GenBank”, UPM with the permission of the proper authority of the Institute of Tropical Agriculture and Food Security, UPM, and the study was implemented in accordance with institutional relevant guidelines and regulations. After continuous selfing, we selected a set of 44 best performing individuals of Bambara groundnut from selfed (fourth) generation (S4). The GPS location of the major growing region of each population and its code and names were listed in Table 1. For DNA extraction fresh leaves were taken from individual plants of each genotype of two weeks aged. The leaf samples were preserved at − 80 °C until the DNA extraction was executed.

**Table 1:** List of Bambara groundnut accessions, population name, code, and their major growing area.

Population	ID	Accession	Population	ID	Accession	Population	ID	Accession
**Pop-1 (Duna)** Lc: Gombe	G1S4	DunP2-18	**Pop-5 (Bidilalle)** Lc: Akko	G17S4	BdilaP10-18	**Pop-9 (Giiwa)** Lc: Gombe	G33S4	GiiwP12-18
	G2S4	DunP8-18		G18S4	BdilaP8-18		G34S4	GiiwP11-18
	G3S4	DunP9-18		G19S4	BdilaP11-18		G35S4	GiiwP9-18
	G4S4	DunP6-18		G20S4	BdilaP5-18		G36S4	GiiwP1-18
**Pop-2 (Maikai)** Lc: Gombe	G5S4	MaikP11-18	**Pop-6 (Jatau)** Lc: Gombe	G21S4	JataP3-18	**Pop-10 (Karu)** Lc: Gombe	G37S4	KarP3-18
	G6S4	Maik12-18		G22S4	JataP5-18		G38S4	KarP10-18
	G7S4	MaikP3-18		G23S4	JataP4-18		G39S4	KarP9-18
	G8S4	MaikP6-18		G24S4	JataP1-18		G40S4	KarP8-18
**Pop-3 (Cancaraki)** Lc: Gombe	G9S4	CancP1-18	**Pop-7 (Maibargo)** Lc: Sokoto	G25S4	MaibP3-18	**Pop-11 (Exsokoto)** Lc: Sokoto	G41S4	ExSokP4-18
	G10S4	CancP2-18		G26S4	MaibP8-18		G42S4	ExSokP3-18
	G11S4	CancP4-18		G27S4	MaibP9-18		G43S4	ExSokP10-18
	G12S4	CancP3-18		G28S4	MaibP6-18		G44S4	ExSokP5-18
**Pop-4 (Roko)** Lc: Kwami	G13S4	RokP6-18	**Pop-8 (Katawa)** Lc: Gombe	G29S4	KataP4-18			
	G14S4	RokP9-18		G30S4	KataP1-18			
	G15S4	RokP1-18		G31S4	KataP5-18			
	G16S4	RokP3-18		G32S4	KataP8-18			

*Pop* population, *Lc* location or area, *G* genotype, *S* selfed generation, *Dun* Duna, *Maik* Maikai, *Canc* Cancaraki, *Rok* Roko, *Bdila* Bidilalli, *Jata* Jatau, *Maib* Maibargo, *Kata* Katawa, *Giiw* Giiwa, *Kar* Karu, *Exsok* Exsokoto.

### Genomic DNA extraction and quantification

For DNA extraction 2.5 g of fresh foliar tissue from the seedling of 14 days of age, healthy and without mechanical damage were used performing the modified protocol of Zheng^28^. Using the mortar and pestle, the presence of liquid nitrogen young healthy leaves tissues was milled into fine powder. The ground leaves tissues were transferred in a 2-mL Eppendorf tube and mixed well with 800 μl of CTAB extraction buffer [100mMTris-HCl (pH 8.0), 20 mM EDTA (pH 8.0), 1.4 M NaCl and 2% CTAB] and 2 μl β-mercapto-ethanol using vortex for 5 min. The suspension was incubated at 65 °C for 60 min at 500 rpm using an electric thermo shaker with continuous gentle shaking. Afterward, an equal volume of chloroform/isoamyl alcohol, CIA (24:1 v/v), was added and the mixture was centrifuged at 4 °C temperature with 14,000 rpm for 10 min to sediment the leaf residues. Collect the supernatant, then transferred into new 1.5 ml tube and add an equal volume of ice-cold Isopropanol and incubated at 4 °C temperature for 30 min afterward centrifuge the tubes again at 14,000 rpm for 5 min to obtain the DNA pellet. To remove the RNA, the RNase enzyme (50 µg/ml) was added and incubated at 37 °C temperature for 60 min. For the purification purpose, ethanol precipitation was done by the addition of potassium acetate (5.0 M) with 2 Vol. of isopropanol at -20 °C temperature followed by shaking for 1–2 min and centrifuged at 13,000 rpm at 4 °C temperature for 10 min. After discarding the top aqueous phase, the DNA pellet was principiated and washed twice by mixing 800 μl 75% ethanol at -20 °C then centrifuged at 13,000 rpm for 10 min, discarded the ethanol, dried at room temperature. The obtained DNA pellet is dissolved in 50 μl of TE buffer (10 mM Tris, 1 mM EDTA pH 8.0). The solution with genomic DNA was measured to check the concentration and quality by the Thermo Scientific NANO DROP Lite Spectrophotometer (THERMO FISHER SCIENTIFIC, Waltham, MA, USA). The quality of DNA extracted was also checked by running the electrophoresis (BIO-RAD, USA) on a 1% agarose gel of DNA samples. To ensure DNA purity, ratio absorbance 260/280 nm and 260/230 nm of more than 1.8 were considered as a standard to proceed for the next steps. A portion of the DNA template was diluted to a final concentration of 40 ng/μl as a working sample for use. Both the stock and diluted DNAs (working sample) were stored at − 80 °C until further use.

### PCR amplification

Initially a total of 96 ISSR primers from “Integrated DNA Technologies Inc.”, Singapore was screened, of which 32 sets of primers were preferred based on their efficacy to detect clear and sharply distinct polymorphic bands across all the 44 Bambara groundnut genotypes (Table 1). Selected 32 ISSR primers used in this study and their properties (sequences, base pair, GC content, melting temperature, and annealing temperature can be found as “Supplementary Table S1” online. PCR reaction mixture contained “2 × Power Taq PCR Master Mix” is 2 × concentration mixture of DNA polymerase, buffer, and dNTP mixture, MgCl, and Taq (BIOTEKE Corporation), primer, template DNA, and nuclease-free water. PCR was performed in T100 Thermal Cycler from BIO-RAD (Hercules, California 94547, USA). The DNA amplification mixture of 25 μL contained: Master mix 12.5 μL, Nuclease free water 8.5 μL, DNA template (40 ng), 2.0 μL, and primer 2.0 μL. The PCR amplification program was carried out using the following conditions of an initial denaturation at 95 °C for 3 min followed by 35 cycles of denaturation at 94 °C for 45 s, annealing at the primer specific temperature for 1 min, extension at 72 °C for 2 min and final extension was adjusted at 72 °C for 10 min and followed by saturated at 4 °C. The amplified products were separated on 1.5% (w/v) agarose gel with 1 × TBE buffer and stained with “GREEN VIEW Nucleic Acid Gel Stain (5.0 μl/100 ml)” by electrophoresis at 80 V for 75 min adjusted with 400A using horizontal gel electrophoresis system (Bio-Rad Wide Mini Sub-Cell GT and Bio-Rad Sub-Cell GT). The gels were photographed and developed under UV light using the Gel Doc XR + documentation system (BIO-RAD, Hercules, CA, USA). We repeated PCR reaction twice with each primer to ensure the primer ISSR marker’s reliability and reproducibility and discarded the primers that showed weakly and no banding pattern. The size of amplified fragments was measured by running a 100 bp DNA ladder (BIOTEKE Corporation) in the gel as a standard size marker.

### Scoring ISSR band

A set of 32 ISSR primers produce a sharp and reproducible band during DNA fingerprinting of 44 Bambara groundnut accessions. Polymorphic ISSR bands were scored by using the UVIDOC software version 99.02 on the manually detecting method for the actual band sizing based on the standard weight of the DNA 100 bp Ladder. The electrophoretic profiles were coded according to the present visible and repeatable bands on the gel-electrophoresis map as “1” and absent of band at the same loci were coded as “0”.

### Statistical analysis

#### Genetic diversity and frequency analysis

For primer data analysis multiple software was used based on the accounted band profiles. Only repeatable, distinct, and well-resolved fragments were coded as presence (1) or absence (0) for each marker and presented as part of a binary matrix. POPGENE version 1.32^37^ was used to calculate genetic diversity for each population such as percent polymorphic bands (PPB), observed number of alleles (*Na*), the effective number of alleles (*Ne*), Nei’s genetic diversity (*H*), Shannon’s information index (*I*). To analyse the genetic diversity in segmented populations, we estimated the total genetic diversity (*Ht*), genetic diversity within a population (*H*s), Nei’s genetic differentiation index among populations (*G*st), where G_ST_ as a function of within and among population heterozygosity *G*_*ST*_ = *(H*_*T*_* − H*_*S*_*)/H*_*T*_ using POPGENE version 1.32. The amount of gene flow between populations (*N*m) was calculated as per McDermott and McDonald^38^ population differentiation [(*Nm* = 0.5 (1 − *Gst*)/*Gst*], using POPGENE version 1.32. To measure the gene frequencies as well as genetic divergence between individuals of Bambara groundnut accessions were also investigated using Nie’s unbiased genetic distances matrix and genetic identities^39^ performing by GENALEX 6.5 software^40^. The analysis of PCA for the 44 V*. subterranea* was carried out using the same data of ISSR primers. The graphical representation based on Euclidian measures of PCA was revealed by NTSYS PC ver. 2.02; PCA biplot was generated using JMP version 16.0 from SAS program, PCA 3D, and pie chart for graphical visualization of eigenvalues and variation ratio for all PCs were illustrated by NCSS 2021 software. Moreover, the scatter matrix with density and box plot for correlation regression study among marker efficiency index (MI, PIC, RP, EMR, H) was visualized by NCSS 2021 software.

#### Genetic relationship analysis

Clustering was performed to determine the relative genetic distance between individuals and to check the consistency of population genetic differentiation. The Nei’s unbiased genetic distance was used to construct a dendrogram or phylogenetic tree for the 11 Bambara groundnut population using UPGMA (Unweighted Pair Group Arithmetic Mean) method in POPGENE program version 1.32 followed by MEGA (Molecular Evolutionary Genetics Analysis) version 6.10 for Windows reported by Tamura et al.^41^ and Nilkanta et al.^42^.

#### Marker efficiency analysis

The performance of the primers was measured by calculating different parameters including polymorphic Information Content (*PIC*), Resolving Power (*RP*), and Discriminating Power (*DP*), expected heterozygosity (*H*), and arithmetic mean heterozygosity (*H*_*avp*_) for each primer by program iMEC (https://irscope.shinyapps.io/iMEC/)^43^. This program calculates PIC using (Botstein et al. (1980) formula *PIC* = 1 – Σ *p*_i_^2^ – Σ Σ *p*_i_^2^ *p*_j_^2^ where p_i_ and p_j_ are the population frequency of the ith and jth allele. The first summation is over the total number of alleles, whereas the two subsequent summations denote all the i and j where i = j. EMR was calculated using Powell et al.^44^ formula *EMR* = *n β*, where n is the average number of fragments amplified by an individual to a specific system marker (multiplex ratio) and *β* is estimated from the number of polymorphic loci (np) and the number of non-polymorphic loci (nnp); *β* = *n*_p_/(*n*_p_ + *n*_np_). The resolving power (RP) of each primer was calculated as Prevost and Wilkinson^45^ formula; *R* = Σ *I*_*b*_, where I_b_ represents the informative fragments. where *Ib* or band informativeness is represented on a scale of 0–1 and is defined as *Ib* = 1 – (2 ×|0.5 – *p*|), where pi is the proportion of individuals containing the ith band. Discriminating Power (DP) estimated by Tessier et al.^46^ as *D* = 1 – *C; where C is* the confusion probability is *C* = Σ *c*i = Σ *p*_i_ N_pi_ − 1 /N − 1 where for *N* individuals, *C* is equal to the sum of all *c*i for all of the patterns generated by the primer. Expected heterozygosity as per formula of* H* = 1 – Σ *p*_i,_ where *p*i is the allele frequency for the *i*th allele, and the summation is over all available alleles. Arithmetic means heterozygosity (*H*_*avp*_) the formula given by Powell et al.^44^ is *H*_avp_ = Σ *H*n/*n*p, where *Hn* is the heterozygosity of the polymorphic fraction of markers and the summation is over all the polymorphic loci *n*p. To characterize the capacity of each primer to detect polymorphic loci among the genotypes, we also calculated de Marker Index (*MI*) for each primer as a product of *PIC* and *EMR*^47^.

#### Genetic structure and admixture analysis

To infer the profile of the population structure and admixture detection, a Bayesian model clustering algorithm by STRUCTURE version 2.3.4^48^ was performed based on ISSRs binary data sets of 44 BG genotypes. Before performing the structure analysis, 44 genotypes were categorized into 1 to 11 distinct population groups (i.e., each population the group comprised of 4 individuals genotype) (Table 1). The Bayesian admixture analysis is one of the most perfect approaches for diploids and polyploids^49^ to sense the patterns of population genetic structure using dominant markers due to it does not assume prior information of inbreeding and Hardy–Weinberg equilibrium even can be executed with a comparatively low population and loci^50^. No pre-data on population origin is necessary to determine the most likely number of populations (K) under the admixture model and correlated allele frequencies^51^. To estimate the best genetic unit, K value, a burn-in period of 5.0 × 10^4^ followed by 1.0 × 10^6^ m Markov Chain Monte Carlo (MCMC) simulations at 4 iterations^52^ with ten autonomous runs were performed with K value was pre-set from 1 to 10^53^. For this purposes the output files of structure analysis were squeezed into a single “Zip-Rar” file then upload online “Structure Harvester 0.6.93 version” (http://taylor0.biology.ucla.edu/structure)^54^ to determine the average Log-likelihood, Ln P(D), probability by K-graph, the most provable K value using ΔK method by Evanno et al.^53^ and Q value (standard Q > 0.60 < Q admixture) showing membership coefficient (%) value. This value assigned accessions to a certain population, finally allocate the accessions into a specific cluster based on the maximum (K) likelihood value was used^49^. The bar plot for best K was documented by STRUCTURE software: Version 2^48^. Moreover, STRUCTURE software: Version 2 also used for calculating the fixation index (F*st*), is the proportional increases of homozygosity. However, the value of the F*st* index of a group of the population can range from “0” (no different) to “1” (completely different) i.e., no alleles held in common^55^.

## Results

### Polymorphism quantification by ISSR primers

**Figure 1:**
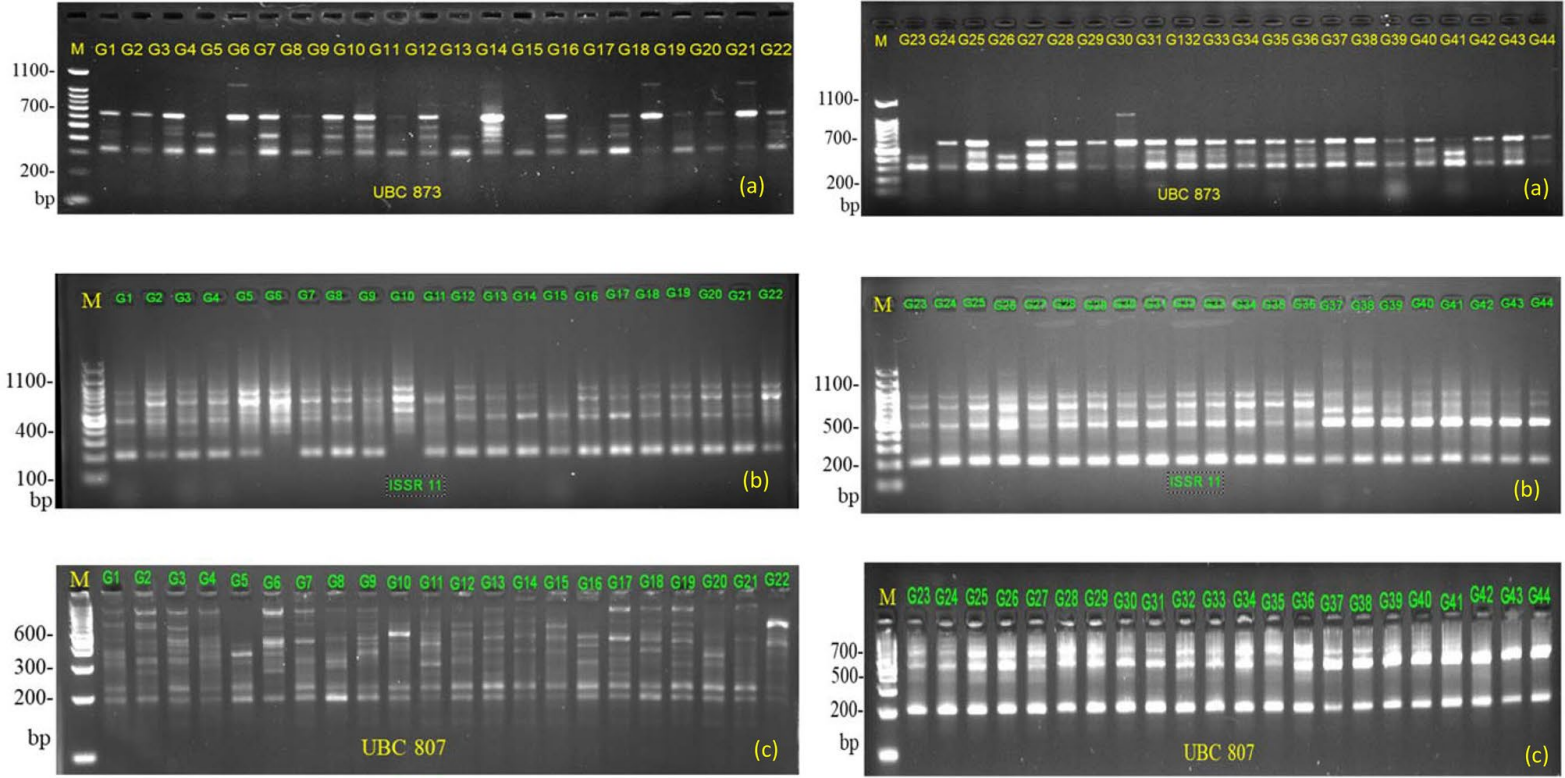
Inter simple sequence (ISSR) banding profiles of 44 V*. subterranea* accessions. The PCR product of **(a)** the UBC 873 primer; **(b)** ISSR 11 primer, and **(c)** UBC 807 amplified with Agarose gel electrophoresis using IMAGE Lab ver. 5.0 program (BIO-RAD). In each gel, we run 22 accessions at a time and lane M refers to 100 bp DNA ladder, and lane with a numeric number refers to the accession number listed in Table 1. The full-length blots/gels are presented in supplementary Fig. S1 online.

The gel image is taken from each primer, based on the result, the gained DNA fingerprinting pattern was very distinct and repeatable (Fig. 1) although detecting the banding pattern and its clarification of specific gel images is always challenging. The range of the amplified band was noted from 100 to 1580 bp (Table 2). Among 96 tested ISSR primers on 44 Bambara groundnut accessions, a set of 32 ISSR primers detected clear and sharp polymorphic bands varied from 11 (UBC 810, ISSR 901, ISSR 842) to 22 (UBC 809, UBC 816, UBC 836) with an average of 15.56 band per primer. However, all the primers generated multiple band patterns spanned from 11 to 22 with a mean of 15.93 alleles per loci. Altogether, out of 510 produced loci, 498 ones were accounts for polymorphic (PPB: 97.64%) indicating the selected primers set was highly efficient for valuation of genetic discrepancy of *V. subterranean* L. accessions (Table 2). The highest percent of polymorphic loci was 100% for the primers (UBC 807, UBC 808, UBC 809, UBC 816, UBC 836, UBC 815, UBC 817, UBC 873, ISSR 811, UBC 835, ISSR 889, ISSR, 812, ISSR 842, ISSR 10, Primer 9, ISSR 856, ISSR 2 M, ISSR 813, ISSR 848 and UBC 825 while the lowest polymorphic percent was observed as 84.62% for UBC 810 primer. The primer ISSR 811 had the highest value of the effective number of alleles (*ne*: 1.59), Nei's gene diversity (*h*: 3.57), Shannon's Information Index (*I*: 0.536) followed by ISSR 10 (*ne*: 1.56; *h*: 0.321; *I*: 0.483) while the lowest was shown by primer UBC 809 (*ne*: 1.26; *h*: 0.2; *I*: 0.345), respectively. The observed number of alleles was recorded low for the primer UBC 810 (*na*: 1.84). The maximum value of gene flow was calculated for primer A-856 (*Nm*: 3.07) followed by ISSR 856 (*Nm*: 2.96) while the least gene flow (*Nm*: 0.47) was observed in ISSR 811 with an average of 2.26 per primer (Table 2).

**Table 2:** The details banding profile and polymorphism revealed by 32 ISSR primers of 44 V*. subterranean* accessions.

Sl No	Marker	na*	ne*	*h*	*I*	TSB	PB	PPB (%)	*Nm*	RABs (bps)
1	ISSR 11	1.95	1.43	0.275	0.43	19	18	94.74	1.48	227–960
2	ISSR 18	1.94	1.3	0.208	0.343	18	17	94.44	1.85	149–889
3	UBC 807	2	1.35	0.226	0.367	17	17	100	2.51	245–960
4	UBC 808	2	1.43	0.276	0.435	16	16	100	2.45	214–590
5	UBC 809	2	1.26	0.2	0.345	22	22	100	2.74	300–900
6	UBC 810	1.84	1.31	0.206	0.331	13	11	84.62	2.83	116–529
7	UBC 816	2	1.28	0.197	0.335	22	22	100	2.96	231–825
8	UBC 836	2	1.48	0.306	0.475	22	22	100	2.93	237–850
9	UBC 841	1.94	1.33	0.228	0.369	19	18	94.74	2.18	210–822
10	UBC 844	2	1.35	0.245	0.398	19	19	100	2.27	136–600
11	UBC-815	2	1.27	0.186	0.312	19	19	100	1.85	207–900
12	UBC 817	2	1.28	0.203	0.343	17	17	100	2.65	294–960
13	UBC 873	2	1.43	0.283	0.447	13	13	100	1.92	341–1050
14	ISSR 811	2	1.59	0.357	0.536	12	12	100	0.47	177–485
15	ISSR 901	1.91	1.36	0.238	0.379	12	11	91.67	1.59	100–1580
16	UBC 835*	2	1.27	0.178	0.299	17	17	100	2.14	379–900
17	ISSR 889	2	1.4	0.244	0.382	15	15	100	2.42	119–485
18	ISSR 812	2	1.41	0.245	0.382	15	15	100	2.40	113–529
19	ISSR 842	2	1.5	0.301	0.457	11	11	100	1.93	228–1286
20	A-856	1.92	1.32	0.215	0.351	14	13	92.86	3.07	279–660
21	I-825	1.93	1.29	0.189	0.311	16	15	93.75	2.96	342–1083
22	ISSR 10	2	1.56	0.321	0.483	12	12	100	1.86	143–436
23	ISSR 17	1.93	1.4	0.259	0.412	15	14	93.33	1.85	308–838
24	Primer 9	2	1.49	0.296	0.457	12	12	100	1.29	359–650
25	ISSR 856	2	1.32	0.218	0.36	18	18	100	2.98	173–640
26	ISSR 2M	2	1.42	0.274	0.434	15	15	100	2.69	182–714
27	UBC 835**	1.94	1.38	0.258	0.41	17	16	94.12	2.54	221–771
28	UBC 813	2	1.4	0.276	0.442	17	17	100	2.11	328–825
29	Primer 3	1.92	1.38	0.246	0.39	14	13	92.86	2.68	196–614
30	ISSR 848	2	1.5	0.326	0.495	15	15	100	1.41	272–800
31	UBC 825	2	1.42	0.259	0.4037	13	13	100	2.83	191–1073
32	UBC 830	1.92	1.32	0.209	0.338	14	13	92.86	2.36	193–613
	Mean	1.97	1.38	0.248	0.395	15.93 (510)	15.56 (498)	97.64	2.26	
	St.Dev	0.145	0.287	0.143	0.187					

*na observed number of alleles, *ne effective number of alleles, *h Nei's gene diversity, *I Shannon's information index; *Nm* estimation of geneflow; range of amplified bands (RABs), *TSB* total scored band, *TSB* total scored band, *PPB* percent polymorphic band.

* and **Both (UBC 835) name are same as per the source of the collection, but the sequences are not same and collected from two different sources.

### Marker efficiency analysis (MEA)

**Figure 2:**
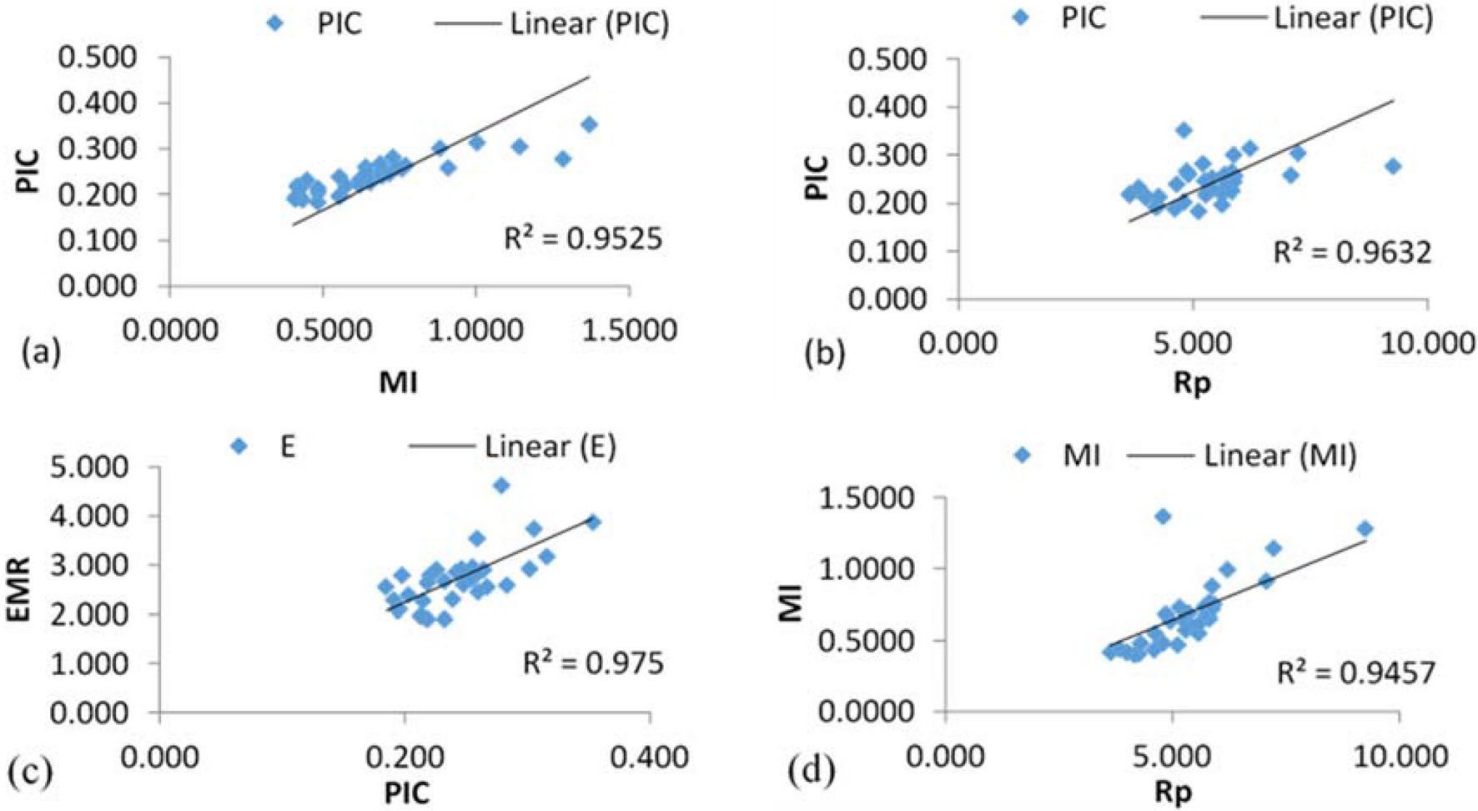
Scatter matrix plot showing relationship **(a)** MI vs PIC, **(b)** Rp vs PIC, **(c)** PIC vs EMR, and **(d)** MI vs Rp generated by ISSR assay.

**Figure 3:**
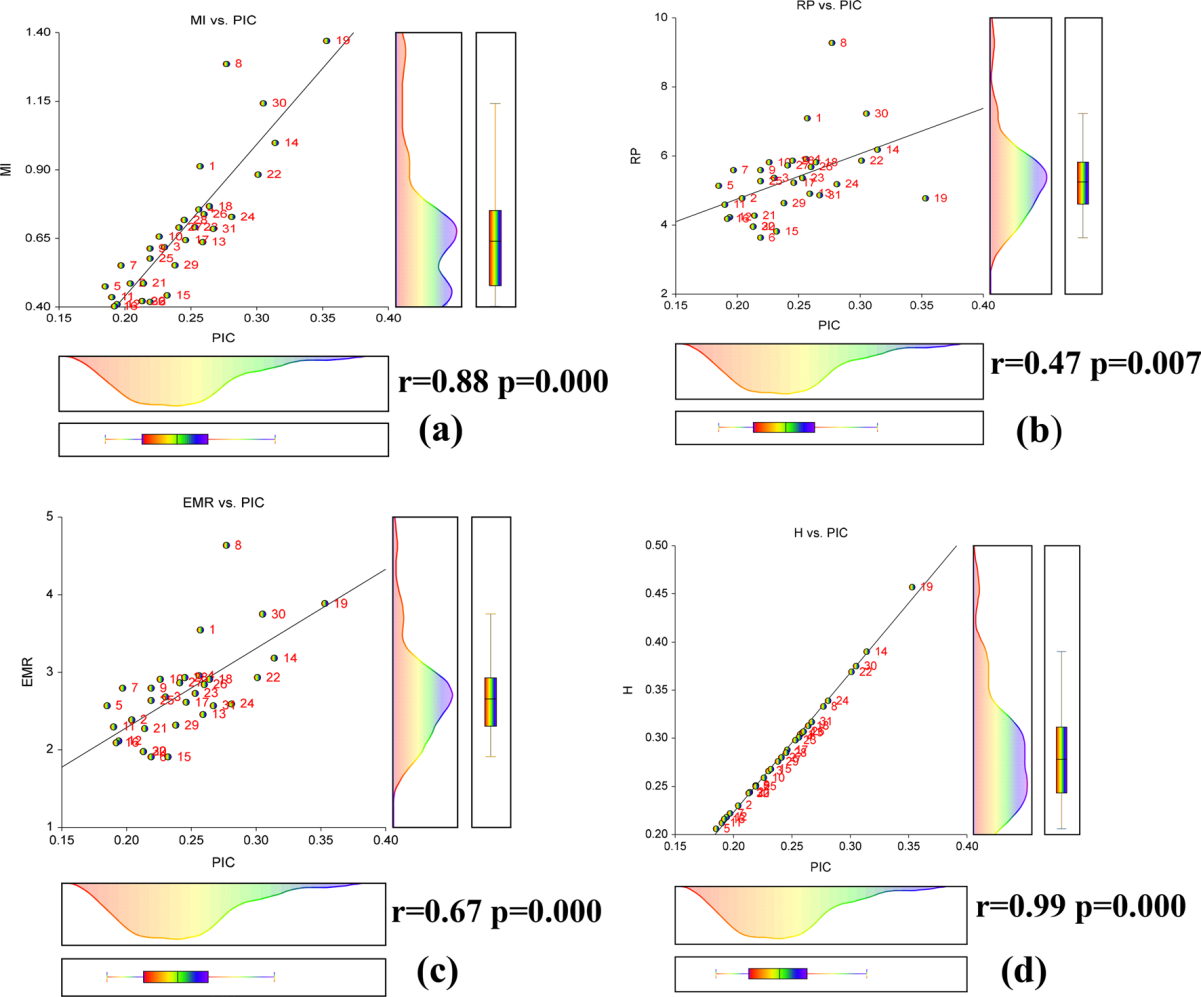
Density plot and box plot showing the relationship and density of primers efficacy. **(a)** MI vs PIC, **(b)** Rp vs PIC, **(c)** PIC vs EMR, and **(d)** H vs PIC generated by ISSR assay.

For calculating the polymorphic efficiency of individual primer *iMEC* is a straightforward trail. Details polymorphic indices of selected ISSR primers are given in Table 3. For each primer, PIC is an indicator of the diversity and frequency of generated alleles among the accessions. On an average of PIC was 0.243 and the highest value was recorded for ISSR 842 (*PIC* = 0.353) followed by ISSR 811 (0.311) whereas lowest was *PIC* = 0.185 for UBC 809. The heterozygosity (*H*) varied from 0.206 (ISSR 809) to 0.457 for ISSR 842 with a mean of 0.285 per primer. The arithmetic means of heterozygosity (*Havp*) ranged between 0.0002 and 0.0009 with the mean of *Havp* = 0.0004 per primer. An extremely conditional factor on the magnitude of primer polymorphism is the effective multiplex ratio (*EMR*) spanned from 1.90 (UBC 810 and ISSR 901) to 4.636 (UBC 836) with an average of 2.71 per primer. The marker index (*MI*) was calculated to recognize the usefulness of the ISSR primer system on Bambara groundnut which was maximum for ISSR 842 (*MI* = 1.37) followed by ISSR 836 (*MI* = 1.28) while least for UBC 835 (*MI* = 0.402) along with a mean of 0.675 per primer. To determine the judicious profundity of primer we calculate discriminative power (*D*) with a mean index of *D* = 0.966 and extended from 0.938 to 0.979. The highest value counted resolving power (*Rp*) is 9.27 for UBC 836 whereas the lowest was *Rp* = 3.63 for UBC 810 with an average of 5.30 per primer. A positive significant correlation (Fig. 2) was found between the PIC vs Rp (r = 0.46, p ≤ 0.05); MI vs PIC values (r = 0.88, p ≤ 0.05); PIC vs EMR (r = 0.67, p ≤ 0.05); as well as MI vs Rp (r = 0.74, p ≤ 0.05). A strong linear relationship was found among MI, PIC, Rp, and EMR index. The regression equations for this linear relationship and coefficient of determination (R^2^) were: (a) PIC = 0.1477 + 0.1405 × MI (R^2^ = 0.95); (b) PIC = 0.1566 + 0.0162 × Rp (R^2^ = 0.96); (c) EMR = 0.2355 + 10.237 × PIC (R^2^ = 0.97); (d) MI = − 0.199 + 0.1648 × Rp (R^2^ = 0.94). The graphical contrasts, scatter matrix plots of MI, PIC, Rp, and EMR for ISSR primer were displayed in Fig. 2. For further elucidation, a density plot and box plot were constructed with each combination of correlation regression relationships, illustrated in Fig. 3.

**Table 3:** Efficacy of primer polymorphism calculated with iMEC of Bambara groundnut genotypes.

Primer	SB (PB)	*H*	*PIC*	*EMR*	*H*avp	*MI*	*D*	*Rp*
ISSR 11	19 (18)	0.304	0.257	3.545	0.0004	0.9129	0.965	7.091
ISSR 18	18 (17)	0.230	0.204	2.386	0.0003	0.4857	0.983	4.773
UBC 807	17 (17)	0.266	0.230	2.682	0.0004	0.6180	0.975	5.364
UBC 808	16 (16)	0.301	0.256	2.955	0.0004	0.7557	0.966	5.909
UBC 809	22 (22)	0.206	0.185	2.568	0.0002	0.4750	0.986	5.136
UBC 810	13 (11)	0.251	0.219	1.909	0.0004	0.4184	0.979	3.636
UBC 816	22 (22)	0.222	0.197	2.795	0.0002	0.5514	0.984	5.591
UBC 836	22 (22)	0.333	0.277	4.636	0.0003	1.2858	0.956	9.273
UBC 841	19 (18)	0.251	0.219	2.795	0.0003	0.6135	0.979	5.591
UBC 844	19 (19)	0.259	0.226	2.909	0.0003	0.6566	0.977	5.818
UBC-815	19 (19)	0.212	0.190	2.295	0.0003	0.4358	0.986	4.591
UBC 817	17 (17)	0.218	0.194	2.114	0.0003	0.4101	0.985	4.227
UBC 873	13 (13)	0.306	0.259	2.455	0.0005	0.6367	0.965	4.909
ISSR 811	12 (12)	0.390	0.314	3.182	0.0007	0.9983	0.930	6.182
ISSR 901	12 (11)	0.268	0.232	1.909	0.0005	0.4425	0.975	3.818
UBC 835*	17 (17)	0.216	0.192	2.091	0.0003	0.4024	0.985	4.182
ISSR 889	15 (15)	0.288	0.246	2.614	0.0004	0.6439	0.970	5.227
ISSR 812	15 (15)	0.313	0.264	2.909	0.0005	0.7674	0.963	5.818
ISSR 842	11 (11)	0.457	0.353	3.886	0.0009	1.3702	0.876	4.773
A-856	14 (13)	0.243	0.213	1.977	0.0004	0.4215	0.980	3.955
I-825	16 (15)	0.244	0.214	2.273	0.0003	0.4864	0.980	4.273
ISSR 10	12 (12)	0.369	0.301	2.932	0.0007	0.8827	0.941	5.864
ISSR 17	15 (14)	0.298	0.253	2.727	0.0005	0.6907	0.967	5.364
PRIMER 9	12 (12)	0.339	0.281	2.591	0.0006	0.7287	0.954	5.182
ISSR 856	18 (18)	0.250	0.219	2.636	0.0003	0.5768	0.979	5.273
ISSR 2M	15 (15)	0.307	0.260	2.841	0.0005	0.7384	0.964	5.682
UBC 835**	17 (16)	0.280	0.241	2.864	0.0004	0.6899	0.972	5.727
UBC 813	17 (17)	0.285	0.245	2.932	0.0004	0.7174	0.970	5.864
PRIMER 3	14 (13)	0.276	0.238	2.318	0.0004	0.5521	0.973	4.636
ISSR 848	15 (15)	0.375	0.305	3.750	0.0006	1.1426	0.938	7.227
UBC 825	13 (13)	0.317	0.267	2.568	0.0006	0.6852	0.961	4.864
UBC 830	14 (13)	0.243	0.213	1.977	0.0004	0.4215	0.980	3.955
Mean	–	0.285	0.243	2.719	0.0004	0.675	0.9669	5.3054

*SB* scored bands, *PB* polymorphic bands, *D* discriminating power, *Emr* effective multiplex ratio, *H* expected heterozygosity, *Havp* mean heterozygosity, *MI* marker index, *PIC* polymorphism information content, *Rp* resolving power.

* and **Both (UBC 835) name is same as per the source of the collection, but the sequences are not same and collected from two different sources.

### Genetic distance (Nei’s measure) analysis

Table 4 disclosed the genetic distances (GD) among 44 V*. subterranea* accessions under 11 populations of 4 geographical zones which spanned from a minimum of GD = 0.14 to a maximum of GD = 0.39. The topmost genetic distance (GD = 0.39) was recorded for the pair of accessions such as G10 vs G25, G10 vs G31, G10 vs G39, G13 vs G32, G13 vs G35, G15 vs G35, and G15 vs G30 from different agroecological zones of Gombe (G10, G35, G30, G31, G32, G39), Kwami (G13 & G15), Sokoto (G25). Although, genotype (G10), genotype (G30, G31, G32), genotype (G35), and genotype (G39) having the same geographical locations but arises from three distinct populations of Cancaraki, Katawa, Giiwa, and Karu, respectively. However, the genotype (G25) comes from Maibergo the population showed less relatedness with the genotype (G10) of Roko. Typically, the accessions with low genetic distance showed extreme genetic identity and vice versa (Table 4). The accession G15 under the population Roko has diverged (GD = 0.39) from the accession G35 from the population Giiwa under the Gombe zones. The second maximum genetic distances GD = 0.38 was accounted for the accessions pair of G10 vs G30, G10 vs G36, G10 vs G44, G12 vs G31, G13 vs G29, G13 vs G30, G13 vs G31, G13 vs G38, G14 vs G32, G14 vs G35, G15 vs G32, G19 vs G38, and G20 vs G38 subsequently the accessions G5 vs G36, G10 vs G32, G10 vs G34, G10 vs G35, G10 vs G38, G10 vs G43, G12 vs G38, G13 vs G25, G13 vs G26, G13 vs G36, G13 vs G37, G13 vs G39, G14 vs G26, G18 vs G35, G19 vs G23, G19 vs G30, G19 vs G31, and G20 vs G43 covered the same genetic distances of GD = 0.37 but emanates from different agro-ecological populations. On the other hand, the accessions G27 vs G28 and G28 vs G29 had the least genetic distance (GD = 0.14) with common population (Maibergo) origin and marked as very closely associated accessions they were though the accessions G28 and G29 come from two distinct the population of Maibergo (Sokoto) and Katawa (Gombe), respectively afterward the genotypes (G21 & G22) under the Jatau population displayed the genetic distance of GD = 0.16 (Table 4).

**Table 4:** Distance matrix (below diagonal) and identity (above diagonal) based on Nei’s original measures for the 44 V*. subterranea* genotypes revealed by ISSR primers.

	G1	G2	G3	G4	G5	G6	G7	G8	G9	G10	G11	G12	G13	G14	G15	G16	G17	G18	G19	G20	G21	G22
G1	***	0.83	0.81	0.82	0.77	0.80	0.78	0.76	0.78	0.75	0.75	0.78	0.76	0.76	0.77	0.79	0.75	0.77	0.76	0.74	0.76	0.78
G2	0.19	***	0.79	0.82	0.78	0.79	0.77	0.77	0.79	0.72	0.75	0.76	0.78	0.76	0.77	0.77	0.75	0.74	0.75	0.74	0.74	0.75
G3	0.21	0.24	***	0.82	0.78	0.80	0.80	0.77	0.76	0.75	0.75	0.76	0.73	0.72	0.74	0.75	0.74	0.74	0.73	0.72	0.73	0.74
G4	0.20	0.20	0.20	***	0.81	0.81	0.77	0.79	0.78	0.75	0.78	0.74	0.74	0.74	0.74	0.75	0.71	0.74	0.73	0.72	0.72	0.75
G5	0.26	0.24	0.25	0.22	***	0.80	0.79	0.78	0.78	0.75	0.76	0.75	0.76	0.74	0.75	0.74	0.73	0.74	0.73	0.73	0.72	0.75
G6	0.23	0.24	0.23	0.21	0.22	***	0.81	0.78	0.79	0.76	0.75	0.74	0.73	0.73	0.75	0.76	0.77	0.74	0.76	0.71	0.75	0.77
G7	0.24	0.27	0.22	0.26	0.24	0.21	***	0.82	0.80	0.76	0.75	0.76	0.76	0.75	0.74	0.76	0.76	0.77	0.74	0.75	0.74	0.76
G8	0.28	0.27	0.26	0.24	0.25	0.25	0.20	***	0.83	0.79	0.78	0.75	0.78	0.77	0.78	0.78	0.79	0.76	0.76	0.75	0.77	0.75
G9	0.25	0.24	0.27	0.24	0.25	0.24	0.23	0.18	***	0.78	0.78	0.80	0.78	0.77	0.78	0.76	0.74	0.75	0.75	0.74	0.76	0.75
G10	0.28	0.33	0.28	0.28	0.29	0.28	0.28	0.24	0.24	***	0.77	0.76	0.77	0.75	0.76	0.76	0.74	0.74	0.72	0.72	0.70	0.71
G11	0.29	0.29	0.29	0.25	0.28	0.29	0.29	0.25	0.25	0.26	***	0.77	0.76	0.78	0.79	0.77	0.74	0.76	0.75	0.71	0.72	0.73
G12	0.25	0.27	0.27	0.30	0.29	0.31	0.27	0.28	0.23	0.27	0.26	***	0.80	0.81	0.81	0.81	0.81	0.78	0.75	0.75	0.77	0.76
G13	0.27	0.25	0.32	0.30	0.27	0.31	0.29	0.25	0.25	0.26	0.27	0.22	***	0.82	0.82	0.80	0.77	0.76	0.74	0.75	0.75	0.75
G14	0.27	0.28	0.33	0.30	0.30	0.31	0.31	0.26	0.26	0.28	0.25	0.21	0.20	***	0.84	0.81	0.77	0.77	0.76	0.77	0.76	0.77
G15	0.26	0.26	0.30	0.30	0.29	0.29	0.27	0.25	0.25	0.28	0.24	0.22	0.20	0.17	***	0.80	0.79	0.78	0.76	0.75	0.75	0.75
G16	0.24	0.26	0.29	0.28	0.30	0.28	0.28	0.25	0.27	0.28	0.26	0.21	0.23	0.21	0.23	***	0.78	0.81	0.78	0.77	0.77	0.79
G17	0.28	0.29	0.30	0.34	0.31	0.26	0.27	0.24	0.30	0.30	0.30	0.21	0.26	0.26	0.23	0.24	***	0.80	0.80	0.77	0.80	0.75
G18	0.26	0.30	0.30	0.30	0.31	0.30	0.30	0.27	0.29	0.30	0.27	0.25	0.27	0.26	0.25	0.21	0.23	***	0.80	0.76	0.78	0.76
G19	0.27	0.29	0.32	0.32	0.31	0.28	0.28	0.27	0.28	0.33	0.29	0.29	0.30	0.27	0.28	0.25	0.22	0.22	***	0.82	0.81	0.78
G20	0.31	0.30	0.32	0.32	0.32	0.34	0.30	0.29	0.31	0.33	0.34	0.29	0.29	0.26	0.29	0.26	0.26	0.27	0.20	***	0.83	0.79
G21	0.28	0.30	0.32	0.33	0.32	0.28	0.27	0.26	0.27	0.35	0.33	0.26	0.29	0.27	0.28	0.26	0.23	0.25	0.21	0.18	***	0.85
G22	0.25	0.28	0.30	0.29	0.29	0.27	0.30	0.29	0.28	0.34	0.32	0.28	0.28	0.27	0.29	0.23	0.28	0.27	0.25	0.23	0.16	***
G23	0.30	0.31	0.31	0.32	0.33	0.34	0.30	0.31	0.35	0.35	0.33	0.30	0.34	0.31	0.36	0.31	0.33	0.32	0.37	0.35	0.33	0.28
G24	0.30	0.28	0.30	0.35	0.35	0.31	0.31	0.32	0.33	0.35	0.32	0.32	0.35	0.32	0.31	0.29	0.29	0.31	0.32	0.34	0.33	0.30
G25	0.32	0.30	0.33	0.35	0.36	0.32	0.34	0.32	0.35	0.39	0.35	0.33	0.37	0.33	0.36	0.30	0.32	0.32	0.36	0.32	0.30	0.29
G26	0.31	0.33	0.32	0.33	0.34	0.33	0.35	0.29	0.36	0.35	0.35	0.35	0.37	0.37	0.35	0.32	0.33	0.31	0.35	0.35	0.32	0.30
G27	0.31	0.32	0.29	0.34	0.35	0.31	0.31	0.29	0.33	0.36	0.35	0.35	0.36	0.34	0.35	0.32	0.30	0.32	0.34	0.33	0.30	0.32
G28	0.32	0.33	0.30	0.32	0.36	0.33	0.33	0.30	0.35	0.34	0.32	0.33	0.34	0.32	0.35	0.32	0.33	0.34	0.34	0.35	0.31	0.29
G29	0.34	0.33	0.31	0.30	0.33	0.30	0.32	0.29	0.34	0.36	0.34	0.35	0.38	0.33	0.35	0.31	0.32	0.33	0.33	0.32	0.29	0.27
G30	0.36	0.33	0.36	0.37	0.36	0.33	0.36	0.35	0.36	0.38	0.34	0.34	0.38	0.36	0.39	0.33	0.34	0.34	0.37	0.35	0.30	0.29
G31	0.33	0.34	0.34	0.33	0.35	0.33	0.33	0.33	0.34	0.39	0.35	0.38	0.38	0.34	0.36	0.32	0.36	0.36	0.37	0.36	0.30	0.31
G32	0.33	0.34	0.34	0.34	0.34	0.33	0.35	0.33	0.34	0.37	0.33	0.35	0.39	0.38	0.38	0.34	0.36	0.36	0.36	0.34	0.28	0.29
G33	0.29	0.32	0.30	0.32	0.33	0.29	0.32	0.30	0.32	0.34	0.30	0.33	0.35	0.31	0.35	0.30	0.31	0.31	0.34	0.31	0.28	0.25
G34	0.33	0.33	0.34	0.35	0.36	0.34	0.35	0.33	0.33	0.37	0.32	0.31	0.34	0.34	0.36	0.31	0.33	0.32	0.36	0.33	0.31	0.31
G35	0.34	0.35	0.34	0.35	0.34	0.32	0.36	0.32	0.37	0.37	0.36	0.35	0.39	0.38	0.39	0.33	0.37	0.37	0.35	0.35	0.32	0.32
G36	0.33	0.33	0.34	0.35	0.37	0.33	0.35	0.37	0.33	0.38	0.35	0.34	0.37	0.35	0.34	0.35	0.33	0.32	0.35	0.34	0.29	0.31
G37	0.34	0.36	0.34	0.33	0.33	0.31	0.32	0.33	0.34	0.34	0.33	0.34	0.37	0.36	0.36	0.33	0.35	0.32	0.35	0.36	0.32	0.31
G38	0.33	0.37	0.35	0.36	0.34	0.33	0.33	0.31	0.35	0.37	0.32	0.37	0.38	0.35	0.35	0.35	0.35	0.31	0.38	0.38	0.33	0.31
G39	0.30	0.31	0.35	0.35	0.33	0.33	0.33	0.33	0.32	0.39	0.35	0.34	0.37	0.36	0.34	0.34	0.33	0.35	0.34	0.36	0.28	0.31
G40	0.34	0.32	0.32	0.33	0.32	0.30	0.32	0.33	0.35	0.35	0.32	0.32	0.32	0.32	0.32	0.30	0.29	0.32	0.35	0.34	0.30	0.28
G41	0.30	0.28	0.33	0.31	0.32	0.32	0.33	0.32	0.31	0.35	0.31	0.31	0.34	0.32	0.32	0.31	0.33	0.33	0.32	0.33	0.32	0.30
G42	0.32	0.32	0.32	0.35	0.34	0.35	0.34	0.31	0.35	0.35	0.34	0.31	0.34	0.24	0.33	0.33	0.32	0.32	0.35	0.32	0.30	0.29
G43	0.30	0.29	0.30	0.31	0.34	0.32	0.31	0.31	0.32	0.37	0.33	0.28	0.32	0.33	0.30	0.28	0.29	0.30	0.31	0.37	0.31	0.31
G44	0.29	0.28	0.32	0.35	0.33	0.30	0.31	0.32	0.31	0.38	0.35	0.29	0.34	0.34	0.29	0.29	0.29	0.31	0.29	0.32	0.28	0.29

	G23	G24	G25	G26	G27	G28	G29	G30	G31	G32	G33	G34	G35	G36	G37	G38	G39	G40	G41	G42	G43	G44
G1	0.74	0.74	0.73	0.74	0.74	0.73	0.71	0.70	0.72	0.72	0.75	0.72	0.71	0.72	0.71	0.72	0.74	0.71	0.74	0.73	0.74	0.75
G2	0.73	0.75	0.74	0.72	0.73	0.72	0.72	0.72	0.71	0.71	0.73	0.72	0.71	0.72	0.70	0.69	0.74	0.73	0.75	0.72	0.75	0.75
G3	0.73	0.74	0.72	0.73	0.75	0.74	0.73	0.70	0.71	0.71	0.74	0.71	0.71	0.71	0.71	0.70	0.71	0.72	0.72	0.72	0.74	0.72
G4	0.72	0.71	0.71	0.72	0.71	0.73	0.74	0.69	0.72	0.71	0.73	0.70	0.70	0.70	0.72	0.70	0.71	0.73	0.73	0.70	0.74	0.71
G5	0.72	0.71	0.70	0.71	0.70	0.70	0.72	0.70	0.71	0.71	0.72	0.70	0.71	0.69	0.72	0.71	0.72	0.73	0.73	0.71	0.71	0.72
G6	0.71	0.73	0.72	0.72	0.73	0.72	0.74	0.72	0.72	0.72	0.75	0.71	0.72	0.72	0.74	0.72	0.72	0.74	0.73	0.71	0.73	0.74
G7	0.74	0.74	0.73	0.71	0.70	0.74	0.72	0.73	0.70	0.72	0.71	0.73	0.71	0.70	0.70	0.73	0.72	0.72	0.72	0.72	0.71	0.73
G8	0.74	0.73	0.73	0.75	0.75	0.74	0.75	0.70	0.72	0.72	0.74	0.72	0.73	0.69	0.72	0.74	0.72	0.72	0.72	0.73	0.73	0.72
G9	0.71	0.72	0.71	0.70	0.72	0.71	0.71	0.70	0.71	0.71	0.73	0.72	0.69	0.72	0.71	0.71	0.73	0.70	0.74	0.71	0.72	0.73
G10	0.71	0.71	0.67	0.70	0.70	0.71	0.70	0.69	0.68	0.69	0.71	0.69	0.69	0.68	0.71	0.69	0.67	0.71	0.70	0.70	0.69	0.68
G11	0.72	0.72	0.70	0.71	0.71	0.73	0.71	0.71	0.71	0.72	0.74	0.72	0.70	0.71	0.72	0.73	0.71	0.72	0.74	0.71	0.72	0.70
G12	0.74	0.73	0.72	0.71	0.71	0.72	0.70	0.71	0.69	0.70	0.72	0.74	0.70	0.71	0.71	0.69	0.71	0.73	0.74	0.73	0.75	0.75
G13	0.71	0.71	0.69	0.69	0.70	0.71	0.68	0.69	0.68	0.68	0.71	0.71	0.67	0.69	0.69	0.68	0.69	0.72	0.71	0.71	0.73	0.71
G14	0.73	0.73	0.72	0.69	0.71	0.73	0.72	0.70	0.71	0.69	0.74	0.71	0.68	0.71	0.70	0.71	0.70	0.72	0.73	0.71	0.72	0.71
G15	0.70	0.73	0.70	0.71	0.71	0.71	0.71	0.68	0.70	0.68	0.71	0.70	0.68	0.71	0.70	0.70	0.71	0.73	0.73	0.72	0.74	0.75
G16	0.73	0.75	0.74	0.73	0.73	0.73	0.74	0.72	0.72	0.71	0.74	0.73	0.72	0.71	0.72	0.71	0.71	0.74	0.73	0.72	0.75	0.75
G17	0.72	0.75	0.73	0.72	0.74	0.72	0.72	0.71	0.70	0.70	0.73	0.72	0.69	0.72	0.71	0.71	0.72	0.75	0.72	0.72	0.75	0.75
G18	0.72	0.73	0.73	0.73	0.73	0.71	0.72	0.71	0.70	0.70	0.73	0.73	0.69	0.72	0.73	0.73	0.70	0.72	0.72	0.73	0.74	0.73
G19	0.69	0.73	0.70	0.70	0.71	0.71	0.72	0.69	0.69	0.70	0.71	0.70	0.70	0.70	0.70	0.68	0.71	0.71	0.73	0.71	0.74	0.75
G20	0.71	0.71	0.73	0.71	0.72	0.71	0.72	0.70	0.70	0.71	0.73	0.72	0.70	0.71	0.70	0.69	0.70	0.71	0.72	0.72	0.69	0.73
G21	0.72	0.72	0.74	0.72	0.74	0.73	0.75	0.74	0.74	0.75	0.76	0.73	0.73	0.75	0.73	0.72	0.75	0.74	0.72	0.74	0.73	0.75
G22	0.76	0.74	0.75	0.74	0.73	0.75	0.76	0.75	0.73	0.75	0.78	0.73	0.73	0.73	0.73	0.73	0.73	0.75	0.74	0.75	0.74	0.75
G23	***	0.82	0.80	0.79	0.82	0.82	0.80	0.80	0.80	0.78	0.78	0.80	0.79	0.77	0.78	0.76	0.78	0.78	0.76	0.79	0.76	0.77
G24	0.20	***	0.83	0.79	0.83	0.80	0.78	0.76	0.79	0.75	0.77	0.78	0.75	0.75	0.74	0.74	0.76	0.74	0.75	0.75	0.75	0.75
G25	0.23	0.18	***	0.80	0.81	0.80	0.79	0.76	0.76	0.79	0.78	0.76	0.76	0.75	0.75	0.73	0.76	0.75	0.74	0.75	0.72	0.74
G26	0.24	0.23	0.23	***	0.82	0.85	0.85	0.79	0.79	0.78	0.82	0.80	0.79	0.76	0.79	0.79	0.75	0.76	0.76	0.79	0.76	0.77
G27	0.20	0.18	0.22	0.19	***	0.87	0.82	0.79	0.81	0.80	0.81	0.79	0.81	0.78	0.78	0.77	0.77	0.77	0.76	0.79	0.78	0.78
G28	0.20	0.22	0.22	0.16	0.14	***	0.87	0.80	0.82	0.81	0.83	0.80	0.81	0.79	0.81	0.81	0.78	0.79	0.77	0.79	0.77	0.78
G29	0.22	0.25	0.24	0.16	0.20	0.14	***	0.84	0.83	0.82	0.84	0.79	0.80	0.79	0.81	0.81	0.80	0.80	0.76	0.78	0.77	0.77
G30	0.23	0.28	0.27	0.23	0.23	0.22	0.18	***	0.83	0.83	0.80	0.80	0.82	0.82	0.82	0.79	0.82	0.83	0.76	0.78	0.77	0.77
G31	0.23	0.24	0.27	0.23	0.21	0.20	0.19	0.18	***	0.83	0.83	0.80	0.80	0.78	0.80	0.81	0.81	0.79	0.78	0.77	0.76	0.77
G32	0.25	0.29	0.23	0.24	0.22	0.21	0.20	0.19	0.18	***	0.83	0.81	0.80	0.78	0.80	0.80	0.82	0.79	0.76	0.78	0.76	0.78
G33	0.25	0.26	0.24	0.20	0.21	0.19	0.18	0.23	0.18	0.19	***	0.80	0.83	0.81	0.80	0.85	0.80	0.78	0.80	0.79	0.78	0.76
G34	0.22	0.25	0.27	0.23	0.23	0.22	0.24	0.23	0.23	0.21	0.23	***	0.802	0.79	0.79	0.80	0.79	0.79	0.79	0.79	0.79	0.77
G35	0.24	0.29	0.27	0.23	0.22	0.21	0.22	0.20	0.22	0.23	0.18	0.22	***	0.81	0.84	0.82	0.81	0.79	0.79	0.80	0.76	0.79
G36	0.26	0.28	0.29	0.27	0.25	0.24	0.23	0.20	0.25	0.25	0.21	0.24	0.21	***	0.84	0.82	0.79	0.84	0.78	0.79	0.77	0.78
G37	0.25	0.31	0.29	0.23	0.25	0.21	0.21	0.19	0.22	0.23	0.22	0.24	0.17	0.17	***	0.83	0.82	0.85	0.79	0.82	0.80	0.80
G38	0.27	0.30	0.32	0.23	0.26	0.21	0.21	0.23	0.21	0.22	0.17	0.23	0.20	0.20	0.19	***	0.85	0.82	0.81	0.80	0.78	0.78
G39	0.25	0.27	0.27	0.28	0.26	0.24	0.22	0.20	0.21	0.20	0.22	0.24	0.21	0.23	0.19	0.17	***	0.83	0.81	0.81	0.80	0.83
G40	0.25	0.30	0.29	0.28	0.26	0.24	0.22	0.19	0.24	0.23	0.25	0.23	0.23	0.18	0.17	0.20	0.19	***	0.79	0.83	0.82	0.83
G41	0.28	0.28	0.31	0.27	0.28	0.26	0.27	0.28	0.25	0.28	0.23	0.23	0.24	0.25	0.23	0.21	0.22	0.24	***	0.81	0.81	0.81
G42	0.24	0.29	0.28	0.24	0.24	0.24	0.25	0.25	0.26	0.25	0.23	0.24	0.22	0.24	0.20	0.23	0.21	0.19	0.21	***	0.83	0.83
G43	0.28	0.29	0.32	0.27	0.25	0.26	0.27	0.27	0.28	0.28	0.25	0.24	0.27	0.26	0.23	0.25	0.22	0.20	0.21	0.18	***	0.84
G44	0.26	0.29	0.30	0.26	0.25	0.25	0.26	0.26	0.26	0.25	0.28	0.26	0.24	0.24	0.23	0.25	0.19	0.19	0.21	0.18	0.17	***

### Genetic relationship

**Figure 4:**
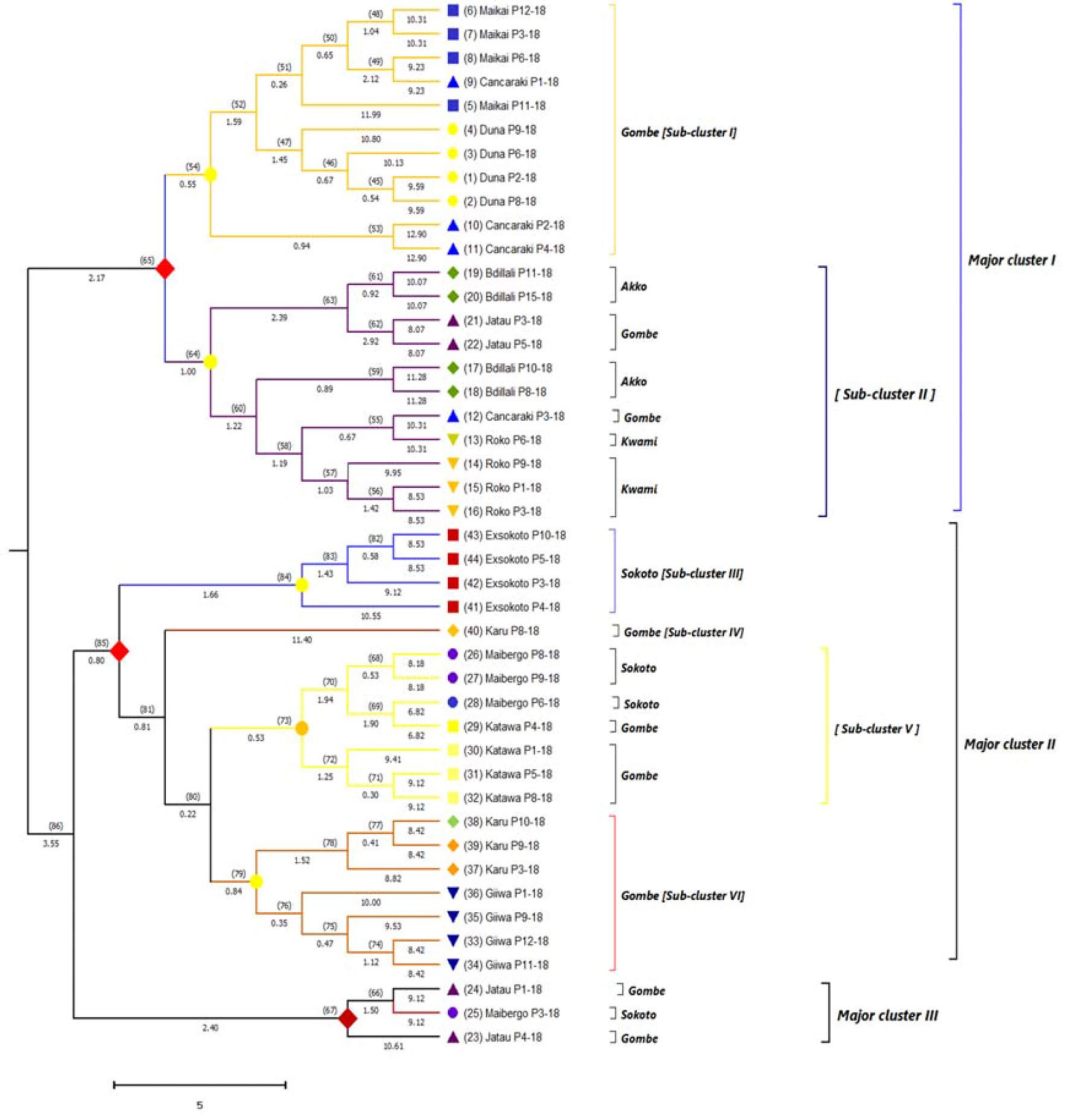
The UPMGA phylogenetic tree illustrating the genetic inconsistency and visual relationship among *V. suberranea* genotypes based on Nei’s genetic distance. In the phylogenetic tree, individual accessions under the same population were marked with similar symbols (circular, square, upward tringle, downward tringle, and diamond shape, etc.) with identical colors. The numeric value beneath branches displayed the edge length (EL) and in the parenthesis upper the branch was node number (NN) while the numeric value in parenthesis behind the accessions name indicates genotype number (Table 1).

The genetic relationship of 44 V*. subterranea* accessions was attained from ISSR primers scoring data set using Nei’s (original) genetic dissimilarity coefficient. The magnitude of relatedness and disparity among the accessions are demonstrated in (Fig. 4 and Table 4). The clustering pattern depicted the accessions into a phylogenetic tree or dendrogram displayed the existence of significant genetic divergence among the evaluated genotypes. The branching pattern of a phylogenetic tree is its topology in which each branch is a line connecting either two internal nodes to each other or an external node to an internal node and the length of a branch denotes the genetic distance. The accessions were expediently grouped into six definite sub-cluster (node remark with yellow-colored circular sign) under three distinct major clusters (node remarked with red-colored diamond shape sign), collected from different geographical origin. The largest among the three major clusters was cluster I which was composed of 22 accessions of different populations emanated from three (Gombe, Akko, Kwami) geographical zones. Eleven of these genotypes originate from the Gombe region positioned into subcluster I with EL = 0.55 and NN = 54, though other genotypes raised from Kwami and Akko collectively with Gombe region comprised of subcluster II with EL = 1.00 and NN = 64. In subcluster I, four accessions of Duna (EL = 1.45; NN = 47) and Maikai (EL = 0.26; NN = 51) positioned into the same group. The genotype Cancaraki P1–18 (EL = 2.12; NN = 49) was separated from Cancaraki P2–18 and Cancaraki P4–18 (EL = 0.94; NN = 53) even though they were in the same subcluster but Cancaraki P3–18 (EL = 0.67 and NN = 55) captured the position at subcluster II. The four genotypes of Roko (Kwami) and Bidillali (Akko), two genotypes of Jatau (Gombe) were assembled into subcluster II where the genotype Bidilalli P11–18 and Bidilalli P15–18 (EL = 0.92; NN = 61) were diverge from Bidilalli P10–18 and Bidilalli P8–18 (EL = 0.89; NN = 59). Under subcluster II, the two genotypes from Jatau (P3–18 and P5–18) showed distinct variation from the other two genotypes of Jatau (P1–18 and P4–18) that positioned into major clusters III. The three accessions of Roko P3–18, Roko P3–18, and Roko _P318_ were in the same group at EL = 1.03 and NN = 57 whereas the genotype Roko P6–18 created a group with Cankaraki P3–18 at EL = 0.67 and NN = 55. All the genotypes from Exsokoto were closely associated with each other and clustered into subcluster III with EL = 1.66 and NN = 84. The lone genotype Karu P8–18 exposed less relatedness among the accessions under major cluster II and isolated into subcluster IV (EL = 11.40) though having a close association with Exsokoto P4–18 (EL = 10.55). The subcluster V (EL = 0.53; NN = 73) accumulated seven genotypes, in which 4 accessions from Katawa (P1–18, P4–18, P5–18, P8–18) and three accessions from Maibergo (P6–18, P8–18, P9–18). The genotype pairs of Maibergo (P9–18 vs P6–18) and Maibergo P6–18 vs Katawa P4–18 exhibited a lower degree of divergence despite their different source of collection such as Gombe and Sokoto. The subcluster VI (EL = 0.84 and NN = 79) compiled the 7 accessions of Giiwa (4 accessions) and Karu (3 accessions) from the same region of Gombe. The accession Giiwa P1–18 (EL = 10) separated from Giiwa P11–18 (EL = 8.42) but closely related with Giiwa P9–18 (EL = 9.53) and Karu P9–18 (EL = 8.82). The accession Jatau P4–18 was noted as genetically divergent from Jatau P1–18 (Gombe) and Maibergo P3–18 although these three accessions collaborate grouped into major cluster III (EL = 2.40; NN = 67).

### Heatmap analysis

**Figure 5:**
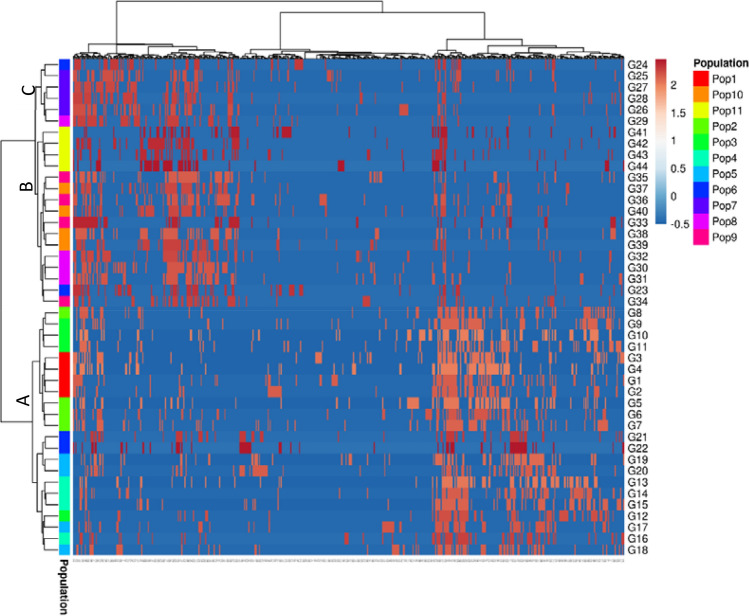
ISSR data-based heatmap of 44 V*. subterranea* accessions constructed by Euclidian distance with Ward (unsquared distances) linkage method using ClustVis Bio tools (https://bio.tools/clustvis). In this plot, the row allocated the 44 accessions under 11 population and the column assign for a total number of loci amplified by ISSR primer.

Based on Euclidian cluster distance and Ward (unsquared distances) linkage clustering method using ISSR data set illustrated three distinct groups of 44 Bambara groundnut accessions (Fig. 5). The genetic relationship study among the accessions revealed by Nei’s distance generated clustering pattern of three major groups which resemble the clustering pattern developed by heatmap analysis. Hence, ISSR linked current research leads to investigating the genetic relatedness among the accessions and identifying the actual genetic distance to avoid any pseudo-diversity. In horizontal dendrogram (rows) represent the accessions and the vertical dendrogram (column) represent the ISSR loci. The red and blue square plots of the heatmap indicate the presence (1) and absence (0) of loci in each accession, respectively. Both rows and columns are clustered using Euclidean distance and Ward (unsquared distances) linkage. Zimisuhara et al.^51^ reported four clusters based on heatmap cluster analysis using ISSR binary data in *Ficus deltoidei* Jack.

### Ordination: principal component (PCA) analysis

**Figure 6:**
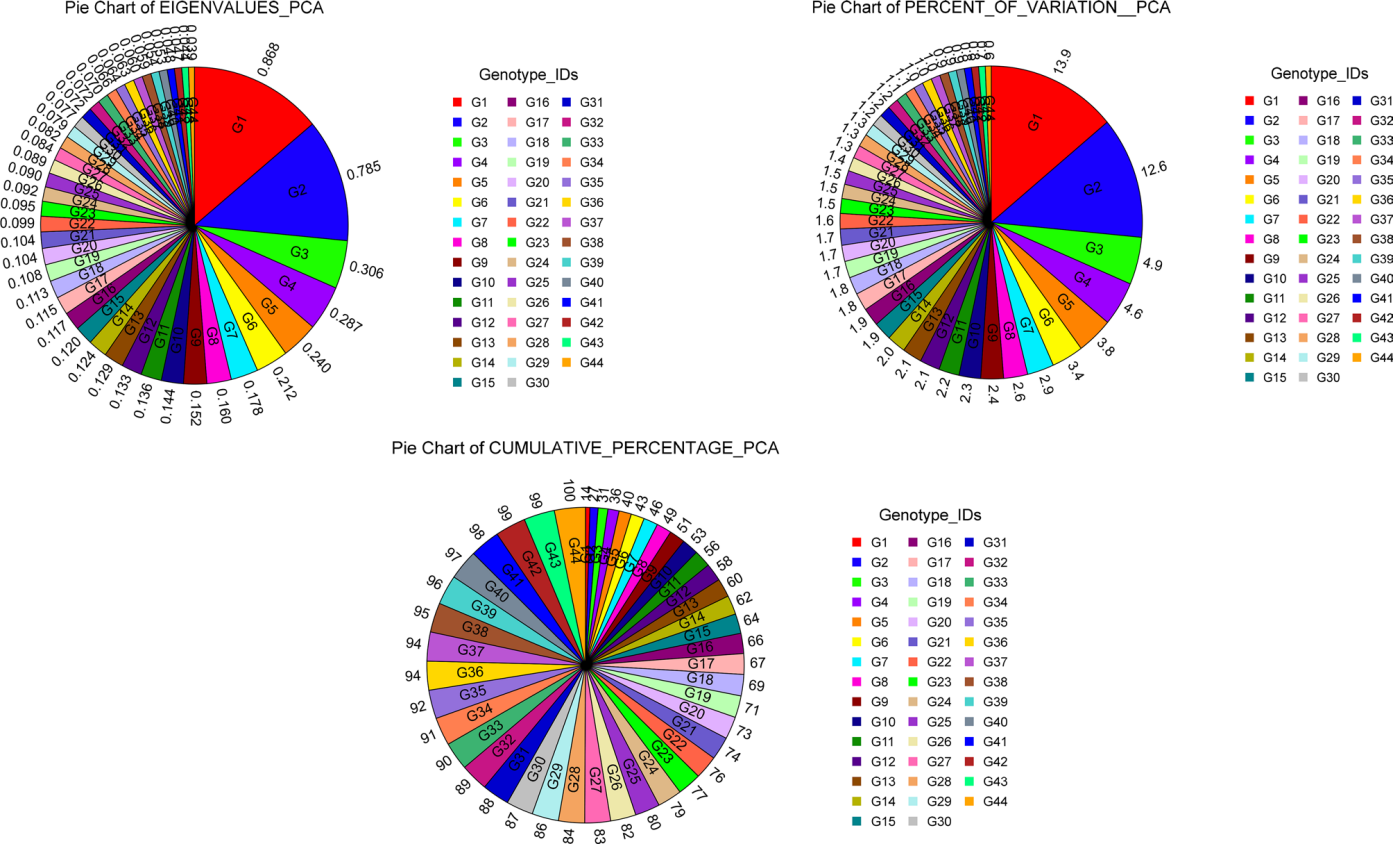
Pie chart showing the eigenvalues, % of variation and cumulative % of variation of 44 axis or PCs revealed by PCA analysis using NCSS 2021.

**Figure 7:**
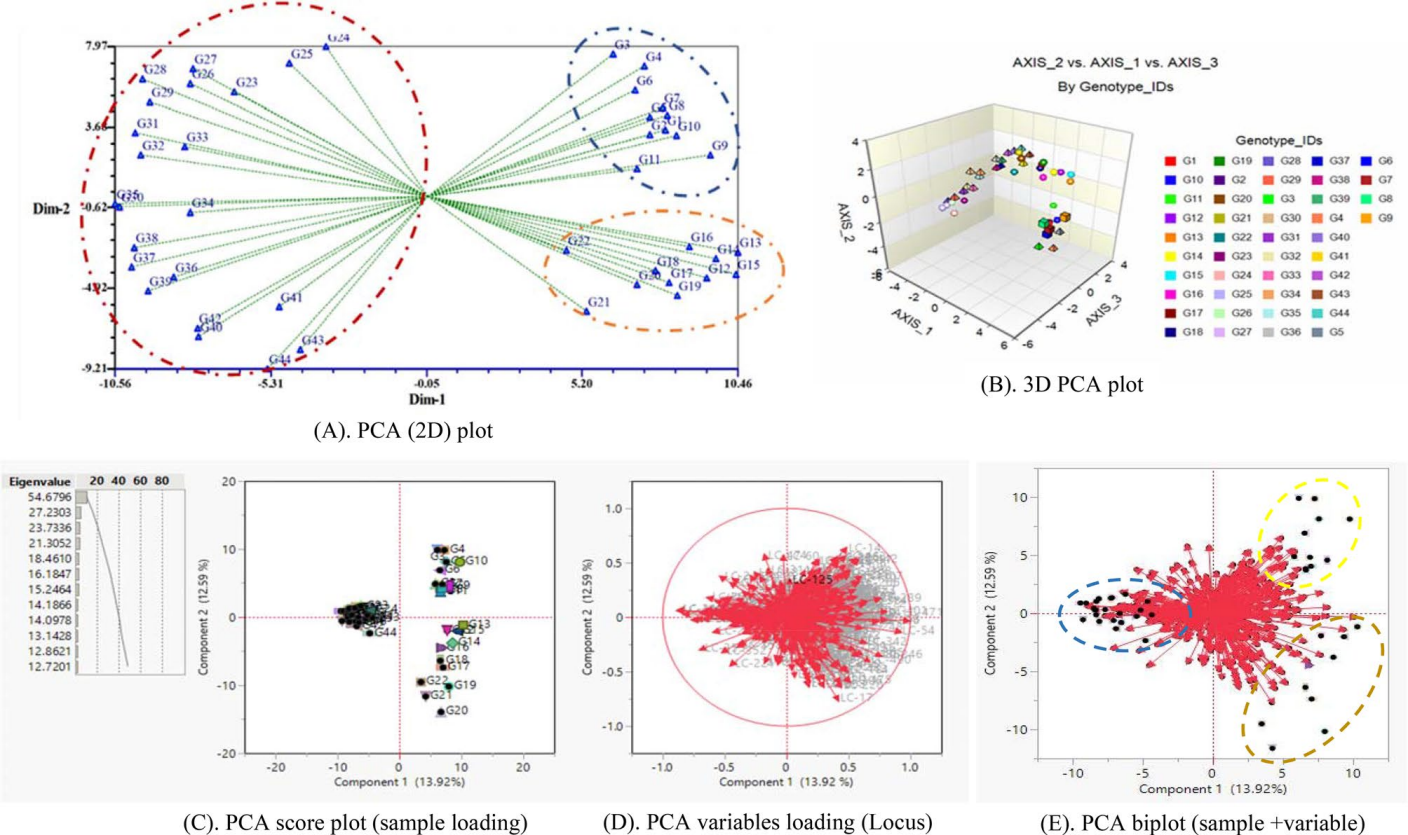
**(A)** PCA (Euclidian’s measure) case scores using NTsys program; **(B)** three-dimensional (3D) graphical display of PCA 3D was generated by using NCSS 2021; **(C)** PCA score plot (sample loading); **(D)** PCA variables loading (loci); and **(E)** PCA biplot representing the loci (red vector) and sample (accessions) loading based on ISSR markers using JMP ver. 16 programs.

Ordination is a collective term for multivariate analysis which adapts a multidimensional group of data in such a way that the similar species or samples are plotted close together while the dissimilar one has placed far apart^56^ also known as multivariate gradient analysis. PCA is used for similarities which starts with the binary data matrix (e.g., presence versus absence of alleles in molecular marker data). When there are no missing data, the output of PCA and PCoA will be similar^57^. To lead the clustering investigation eigenvalues and total percentages of principal component case scores were used. The graphical distribution of eigenvalues, percent of genetic variation, and cumulative percent of genetic variation based on all axes (PCs) were displayed by pie chart in Fig. 6. The first three principal components covered 31.42% (PCA) of cumulative variation (Table 5) and which is accounted for greater than the total variation exposed in the populations. However, 51.12% total variation was captured by 1^st^ nine principal components as shown in Table 5. The PCA analysis revealed that first three principal components captured PC1 = 13.92%, PC2 = 12.59% and PC3 = 4.91% of total variation. Moreover, the PCA analysis has 44 principal components (PCs) out of which the first 25 PCs and 10 PCs contributed 80% and 53% of the total variation (Fig. 6). In the case of PCA analysis from the principal component one (PC1), the highest value was 0.25 for the accessions (G28, G29, G35, G38) followed by 0.24 for the accessions (G30, G31, and G37) while the least values (0.00) were found for the accessions G10 and G19 which have no contribution to total diversity (Table 5). Furthermore, in PC1, most of the accessions contributed positively toward the variation of one group than another except accessions G12, G13, G14, G15, and G20 bearing negative values. In PC2, 17 accessions and 22 accessions in PC3 had a positive contribution to diversity (Table 5). Two dimensional (2D: Fig. 7A) and three dimensional (3D: Fig. 7B) visual illustration of PCA analysis exposed that the entire accessions were distinctly grouped into three genetic components based on Euclidian distance which is the evidence of findings of clustering pattern analysis. In the PCA plot, we observed that within and among the accessions genetically associated genotypes were placed closer to each other while the distant genotypes were positioned far apart. Most of the accessions exhibited similar values of Shannon diversity (Hˊ indices) with a range from 1.86 to 2.01. The highest value was 2.01 for the accessions (G4 and G5) afterward 2.0 for accession (G10) while the least was 1.86 recoded for accessions G22 and G44 (Table 5).

**Table 5:** Hˊ index, eigenvalues and percentage of total variation contributed by principal component (PCs).

Axis	Principal component (PC)									Shannon diversity (*Hˊ* indices) (Log base 10 method)
	PC1	PC2	PC3	PC4	PC5	PC6	PC7	PC8	PC9	
Eigenvalues	0.87	0.79	0.31	0.29	0.24	0.21	0.18	0.16	0.15	
% of variation	13.92	12.59	4.91	4.60	3.84	3.41	2.85	2.56	2.43	
Cum. % of variation	13.92	26.51	31.42	36.02	39.87	43.27	46.12	48.68	51.12	
G1	0.04	−0.24	−0.07	0.12	−0.07	0.23	−0.24	0.15	−0.13	1.95
G2	0.04	−0.22	−0.06	0.15	−0.04	0.28	−0.32	0.02	0.08	1.96
G3	0.06	−0.2	−0.17	0.21	−0.09	0.18	0.05	0.02	−0.21	1.98
G4	0.06	−0.24	−0.14	0.26	−0.14	0.04	−0.17	0.12	−0.13	2.01
G5	0.03	−0.21	−0.07	0.23	−0.14	−0.04	−0.07	−0.10	0.00	2
G6	0.04	−0.21	−0.12	0.20	−0.25	0.05	0.12	−0.05	−0.13	1.95
G7	0.03	−0.22	−0.1	0.15	−0.13	0.05	0.29	−0.23	0.11	1.96
G8	0.05	−0.24	−0.09	0.06	−0.02	−0.14	0.32	−0.01	0.29	1.95
G9	0.01	−0.24	−0.02	0.14	−0.03	−0.13	0.02	−0.09	0.35	1.96
G10	0.00	−0.2	−0.07	0.12	0.14	−0.27	0.18	−0.05	−0.09	2.01
G11	0.02	−0.19	−0.01	0.10	0.16	−0.29	−0.02	0.12	−0.01	1.97
G12	−0.01	−0.22	0.13	−0.08	0.25	0.01	−0.09	−0.18	−0.06	1.93
G13	−0.01	−0.24	0.1	−0.05	0.24	−0.11	−0.15	−0.12	0.04	1.99
G14	−0.01	−0.23	0.08	−0.16	0.24	−0.18	−0.23	0.01	0.08	1.96
G15	−0.01	−0.25	0.14	−0.05	0.25	−0.06	−0.04	0.01	0.09	1.97
G16	0.01	−0.22	0.06	−0.17	0.13	−0.01	−0.13	0.08	−0.20	1.92
G17	0.01	−0.21	0.11	−0.21	0.06	0.09	0.33	−0.10	−0.05	1.95
G18	0.02	−0.2	0.1	−0.19	0.06	0.00	0.12	0.18	−0.28	1.95
G19	0.00	−0.2	0.12	−0.23	−0.18	0.12	0.20	0.19	0.06	1.97
G20	−0.01	−0.16	0.07	−0.33	−0.23	0.02	0.00	0.07	0.15	1.96
G21	0.03	−0.15	0.12	−0.34	−0.37	0.02	0.02	−0.11	0.12	1.9
G22	0.01	−0.12	0.03	−0.25	−0.32	0.01	−0.29	0.00	−0.16	1.86
G23	0.18	0.01	−0.19	−0.05	0.18	0.12	−0.11	−0.30	−0.05	1.9
G24	0.13	−0.02	−0.28	−0.13	0.24	0.26	0.00	−0.04	0.11	1.91
G25	0.14	0.00	−0.27	−0.20	0.09	0.19	−0.10	−0.12	0.08	1.93
G26	0.22	0.01	−0.19	−0.09	0.08	0.03	0.15	0.28	−0.12	1.94
G27	0.23	−0.01	−0.22	−0.11	0.11	0.15	0.20	0.01	0.08	1.93
G28	0.25	0.02	−0.2	−0.10	0.12	−0.02	0.08	0.13	−0.04	1.91
G29	0.25	0.01	−0.15	−0.10	−0.08	−0.10	0.08	0.07	−0.10	1.91
G30	0.24	0.05	0.02	−0.06	−0.06	−0.13	−0.09	−0.29	−0.14	1.94
G31	0.24	0.04	−0.1	−0.03	−0.04	−0.11	−0.13	−0.03	0.17	1.94
G32	0.22	0.05	−0.07	−0.04	−0.14	−0.11	−0.13	−0.15	0.19	1.93
G33	0.23	0.00	−0.07	−0.05	−0.09	−0.20	−0.09	0.32	0.02	1.88
G34	0.2	0.03	0.02	−0.04	0.12	−0.03	−0.10	0.01	0.10	1.92
G35	0.25	0.05	0.03	0.03	−0.08	−0.10	0.01	0.05	0.00	1.95
G36	0.22	0.02	0.15	0.00	−0.05	−0.09	−0.03	−0.11	−0.21	1.95
G37	0.24	0.04	0.16	0.11	−0.04	−0.15	0.11	−0.09	−0.23	1.92
G38	0.25	0.03	0.12	0.08	−0.04	−0.22	0.03	0.20	0.04	1.96
G39	0.22	0.04	0.18	0.09	−0.08	0.05	−0.09	−0.14	0.29	1.91
G40	0.23	−0.01	0.26	0.06	0.00	0.00	0.00	−0.28	−0.23	1.93
G41	0.14	0.01	0.21	0.16	0.05	0.07	−0.14	0.36	0.26	1.88
G42	0.19	0.03	0.23	0.06	0.08	0.17	0.08	0.04	0.02	1.89
G43	0.14	−0.01	0.28	0.14	0.14	0.28	0.10	0.12	−0.04	1.89
G44	0.15	0.01	0.3	0.07	0.01	0.35	0.11	−0.03	0.10	1.86

*PC* principal component, *CUM* cumulative.

#### Biplot analysis

The biplot-based representation of PCA showed the association of evaluated 44 accessions of Bambara groundnut along with ISSR loci loaded in the same plot. The principal component analysis assembled the total tested accessions in a diverse group based on the ISSR data set. Using the JMP version 16.0 analytical tools from the SAS program, we generated PCA sample (accessions) loading (Fig. 7C), PCA variables (Loci) loading (Fig. 7D) which revealed the exact variation among accessions and ISSR loci. The PCA biplot (Fig. 7E) displayed the distribution of entire populations into three distinct groups (Fig. 7E) based on PC1 (13.92%) and PC2 (12.59%). Among the 44 accessions, the accessions G34, G36, G30, G37, G38, G39, G40, G41, G42, G43, and G44 positioned on the negative quartile of the PC1 and PC2 while other accessions were placed into the positive side of PC1 and PC2. In PCA biplot red vector indicates the position of ISSR loci and the different indicator (colored) represent individual accessions were placed in the same plot. The nearly placed accessions in the PCA plot suggesting the accessions are highly correlated and vice versa. Zimisuhara et al.^51^ reported a similar type of PCA biplot-based analysis using ISSR primers in *Ficus deltoidea* Jack.

### Admixture analysis

**Figure 8:**
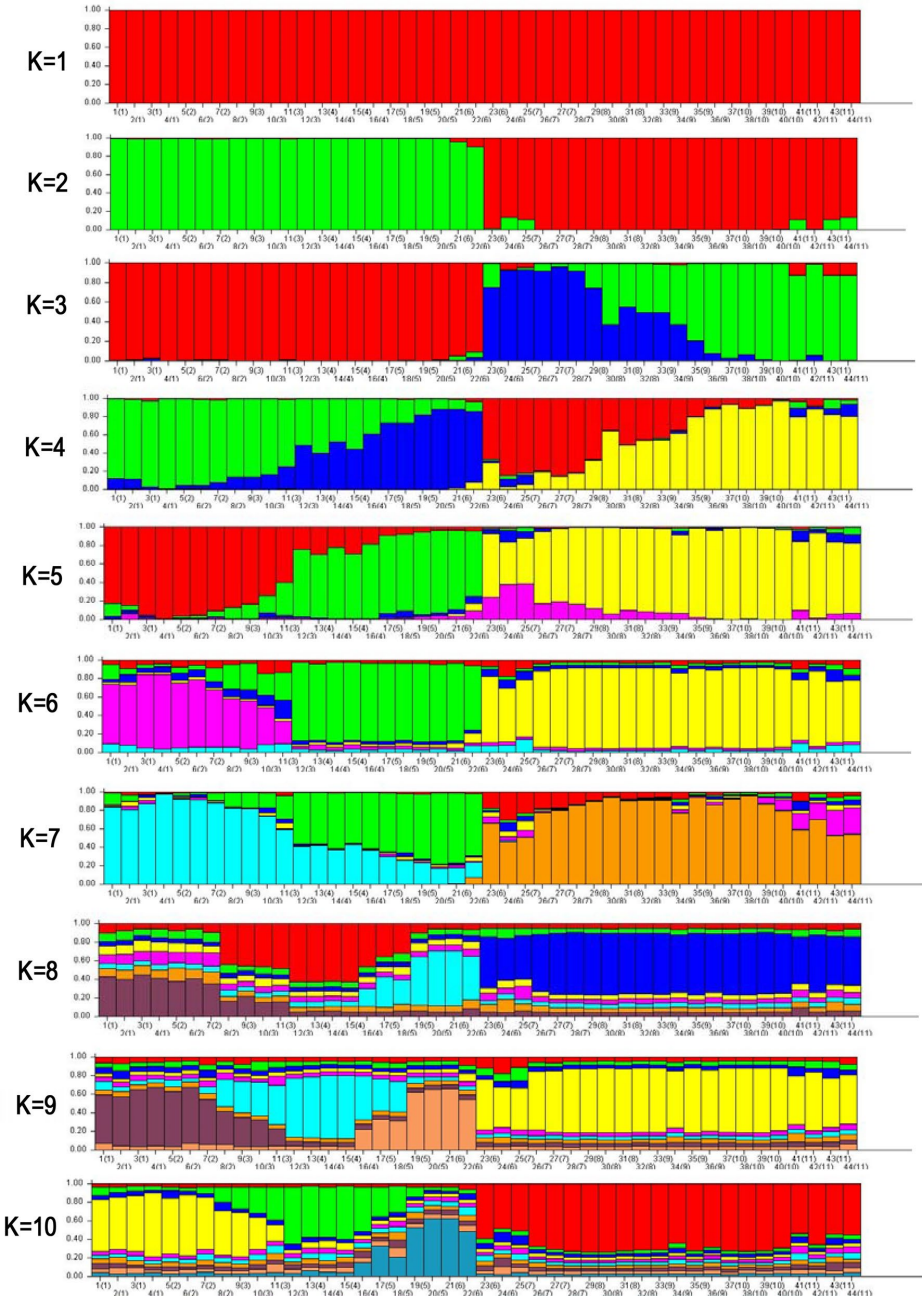
The population membership of the studied Bambara groundnut species group for a priori distinct number of K = 1–10 inferred by the STRUCTURE software (PRITCHARD LAB, CA, USA). Each accession is signified by a vertical column divided into colored segments that represent the individual’s estimated association fractions in K clusters and black vertical lines isolated the 44 accessions. The numeric number beneath the adjacent bar graph indicates the accession IDs and source of sampled population (11) code in parenthesis that is mentioned as in Table 1.

**Figure 9:**
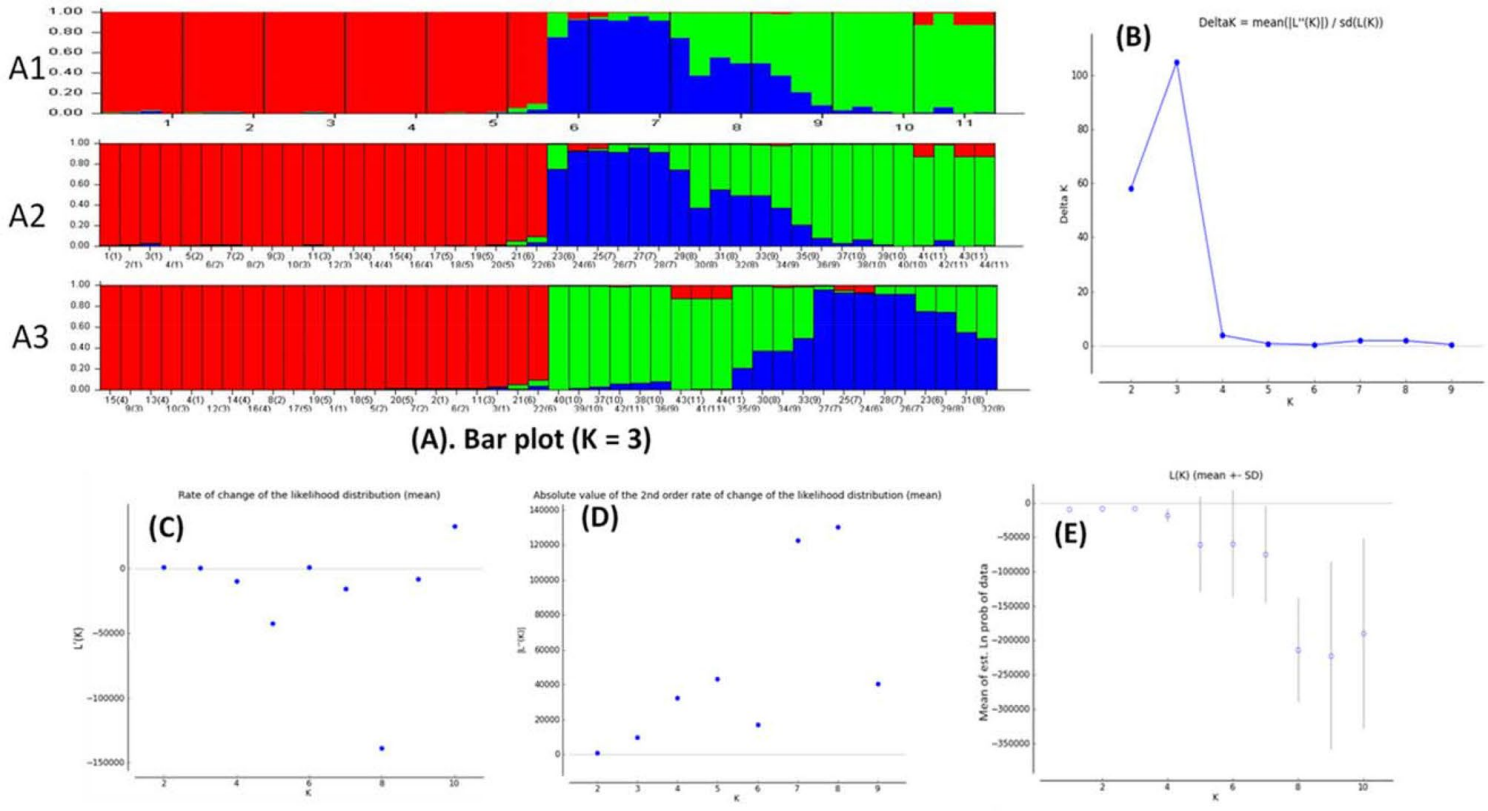
Structure harvester and Delta K value elucidated using Evano et al.^53^ method and Bayesian model-based valuation of population structure for 44 V*. subterranea* accessions based on ISSR markers. **(A)** Bar plot: **(A1)** based on original population order, **(A2)** based on genotype ID (population) order, **(A3)** based on estimated membership coefficients values (Q); **(B)** ΔK = mean (|L”(K)|)/sd(L(K)) here, ΔK = 3 indicates the maximum K value; **(C)** rate of change of the likelihood distribution (mean); **(D)** absolute value of the 2nd order rate of change of the likelihood distribution (mean); **(E)** mean of estimated Ln probability. Each vertical line represents an accession and different color represents the estimated membership coefficients (Q) to the respective group. The red, purple, and yellow colors represent the members of 3 groups or clusters inferred by STRUCTURE harvester. The numbers at the base of each vertical line indicate the accession ID numbers according to the Table 1. The values inside the parentheses indicate the eleven species sources as follow: 1, 2, 3, 4, 5, 6, 7, 8, 9, 10 and 11 represents the Duna, Maikai, Cancaraki, Roko, Bidillali, Jatau, Maibergo, Katawa, Karu and Exsoskoto, respectively.

The structure is a population analysis tool used to assess the patterns of genetic structure from a set of samples. To identify subsets of the whole sample by detecting allele frequency differences within the data and can assign individuals to those sub-populations based on analysis of likelihoods. The structure uses data from individuals in a population to identify allele frequency differences. The genetic structure of accessions was estimated based on Bayesian (theorem) clustering analysis using the STRUCTURE program of Evanno et al.^53^ method followed by Structure harvester. The structure analysis *of V. subterranea* accessions was initially performed based on the maximum number of (K = 1 to 10) as the original population order displayed in Fig. 8. However, the most probable value of population was calculated to the maximum peak at ΔK = 3 (K value = 104.97; Lnprob (K) = − 8053.2) (Fig. 9B) with a rate of change of the likelihood distribution (mean) (Fig. 9C); Absolute value of the second order rate of change of the likelihood distribution (mean) (Fig. 9D) and mean of estimated Ln probability in Fig. 9E. Based on best K = 3 determining that all the evaluated accessions might be positioned into three major clusters visualized with three distinct colors of red, yellow, and purple (Fig. 9A). Determination of delta K is an ad hoc quantity related to the second-order rate of change of the log-likelihood of data related to the number of clusters^51^. Nevertheless, regarding the membership likelihood (Q) > 0.60, some of the accessions showed unique standards which lead to the pure population while Q < 0.60 regarded as the admixture populations^49^. In the bar plot (Fig. 9A), 22 accessions under the red color zone were recorded as highly pure ones whereas the other 6 accessions and 16 accessions were assembled in the yellow and purple color zone, respectively represented as admixture units. Based on Q > 0.60 as the purity standard, in the yellow zone, 6 accessions (G40, G39, G37, G43, G41, and G44) declared as pure ones though G43, G41, and G44 received genetic material from the population of the red zone. Moreover, out of 16 accessions of purple color zone 9 accessions (G42, G38, G36, G35, G30, G34, G33, G31, and G32) were noted as admixture units based on Q < 0.60 (Fig. 9A).

### Fixation index (F_*ST*_ values) analysis

**Figure 10:**
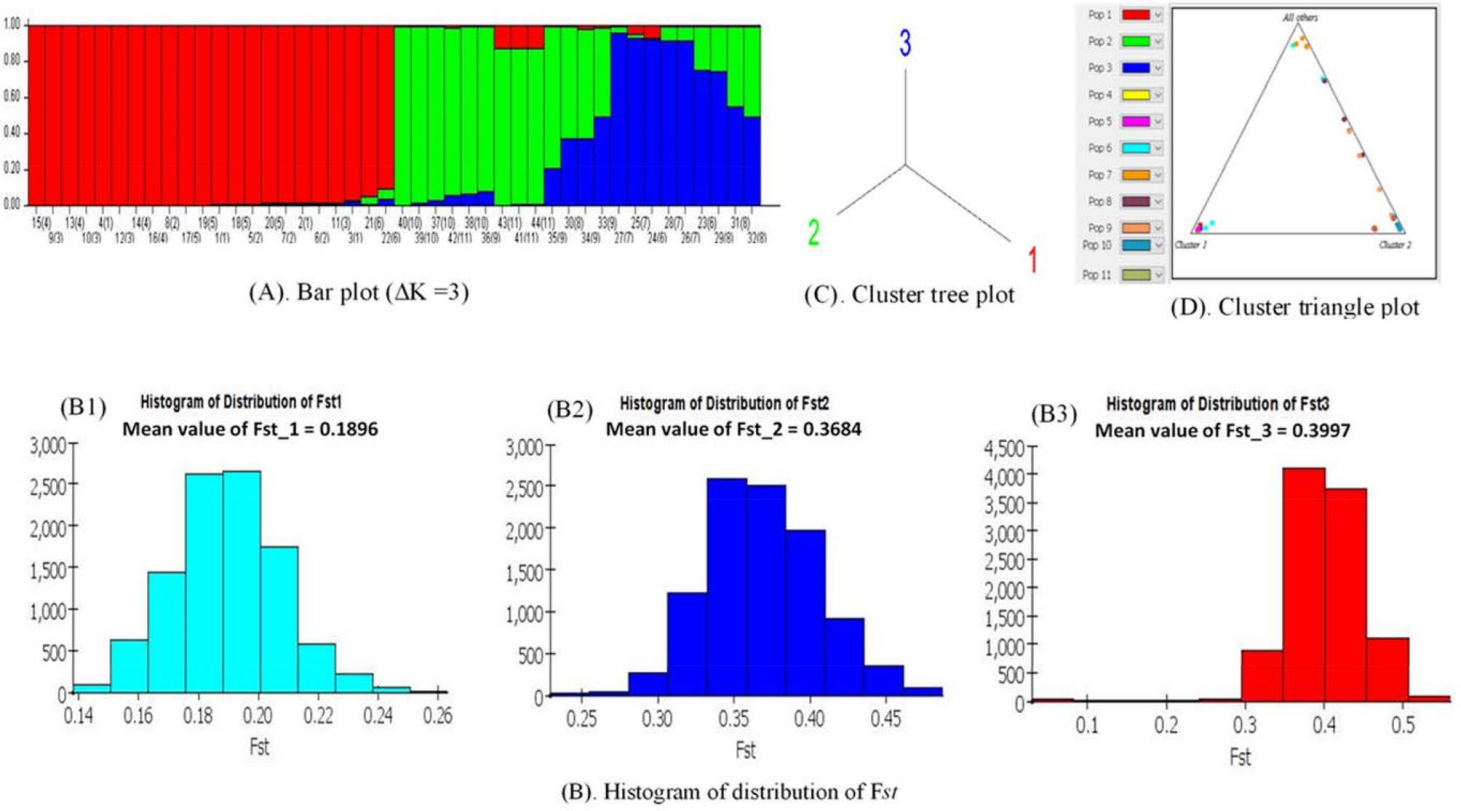
The STRUCTURE software (PRITCHARD LAB, CA, USA) based illustration of fixation index (Fst): **(A)** bar plot based on ΔK = 3; **(B)** histogram of distribution of fixation index (**B1** F*st* mean value for cluster 1, **B2** F*st* mean value for cluster 2, **B3** F*st* mean value for cluster 3); **(C)** tree plot of three cluster; **(D)** triangular plot showing distribution of 11 population by three clusters.

Measuring gene flow (also called gene migration or allele flow) can further be accelerated by the estimation of F*st* (also known as Fixation index)^58^. Genetic differentiation among the accessions due to genetic structure is measured by the fixation index (Fst) using genetic polymorphism data. It is one of the most frequently applied statistics in explaining the population genetic structure^59^. Using the STRUCTURE program, the fixation index can be measured. Considering the best delta K value (ΔK = 3), resultant the entire genetic component is grouped into three clusters. The Structure output can be displayed as a “triangle plot” in which two clusters were plotted at two vertices and all others were at the third. In the triangle plot (Fig. 10D), individuals are represented by a colored dot that corresponds to the different populations. Individuals who are in the corners are assigned to one population or another. The distance between each cluster was shown in Table 6 and the maximum distance was recorded for cluster 1 vs cluster 2 (0.066) followed by cluster 1 vs cluster 3 (0.065), whereas cluster 2 and 3 (0.05) were closely associated with each other displayed by the tree plot (Fig. 10C). Average distances (expected heterozygosity) between individuals in the same cluster were recorded highest for cluster 1 (0.2478). The graphical distribution of the F*st* means the value of three clusters was displayed in Fig. 10B. The estimated mean F*st* value (Table 6) for the accessions under cluster 1 was F*st*_1 = 0.1896 (Fig. 10 B1) while cluster 2 had F*st*_2 = 0.3684 (Fig. 10 B2) and F*st*_3 = 0.3997 for cluster 3 (Fig. 10 B3). Bar plots can be used to further clarification of gene flow between individuals (Fig. 10A). Here, each accession is represented as a unique bar on the bar plot and separated by a black vertical line. Moreover, observing the membership coefficient (Q > 6.0) in the bar plot, the accession G21, G22, G3 in cluster 1 (red) has gene flow from the population of cluster 2 (yellow) and cluster 3 (purple). Accessions (G37, G42, G38, and G36) have gene flow from the cluster 3 population. Accessions G43, G41, G44 have gene flow from the population of cluster 1 (red). Accessions (G30, G33, G35, G34, G27, G28, G26, G23, G29, G31, and G32) have gene flow from the population of cluster 2 (yellow) while accession G24 and G25 received genetic material from the population of cluster 1(red).

**Table 6:** Allele-freq. divergence among pops (Net nucleotide distance), computed using point estimates of P through structure analysis.

Cluster No	Cluster 1	Cluster 2	Cluster 3	AD^a^	Mean value of Fst
Cluster 1	0			0.2478	0.1896
Cluster 2	0.0663	0		0.1966	0.3684
Cluster 3	0.0651	0.051	0	0.1956	0.3997

^a^Average distances (expected heterozygosity) between individuals in same cluster.

## Discussion

### Polymorphism quantification by ISSR primers

The use of a molecular marker is a very common phenomenon to investigate the population structure and genetic diversity as well as distinguishing one genotype from another as a prerequisite for pre-breeding and breeding of crops improvement. Molecular genetic diversity is very crucial as it gives a greater precise measure of polymorphism related to morphological characterizations. Most of the markers showed complete polymorphism suggesting the efficacy of these ISSR markers for the assessment of genetic variation among the *V. subterranea* species. Usually, the efficiency of a certain primer for evaluating the population genetic structure is extremely subjected to the level of polymorphism that could be generated among the accessions. Our results represented a moderate to a higher level of genetic diversity among the studied accessions. A similar trend of diversity is very common as self-pollinated members of Bambara groundnut from the genus *Vigna*, recommend its medium genetic base, which is perhaps assembling of novel gene incorporation due to dynamic forces of natural selection. In this current study, the magnitude, and pattern of genetic variation within 44 V*. subterranea* accessions using 32 ISSR primers exposed the availability of polymorphism. We detected a total of 510 DNA fragments with an average of 15.93 bands per prime. In the present study, comparatively high percentages of polymorphism (97.64%) were observed using the ISSR primer serve as a high potential tool for genetic discrimination among the closely related *V. subterranea* species. Relative studies in *vigna* species particularly in *V. subterranea* based on RAPD, AFLP, SSR, DArT array, and ISSR primer systems were effectively used and reported by researchers (Massawe et al.^16^; Massawe et al.^14^; Ntundu et al. 2004^17^; Amadou et al.^13^; Somta et al.^19^; Rungnoi et al.^1^; and Odongo et al.^22^. However, the reports on *V. subterranea* using ISSR are very insufficient. Only the result by Rungnoi et al.^1^ reported, mean genetic diversity and Shannon diversity in *V. subterranea* as 0.179 and 0.227, respectively using ISSR and RAPD which is lower compared to our findings; Odongo et al.^22^ using SSR reported average percent of polymorphic bands 79. 83 (%); Ntundu et al.^17^ reported a total of 49 polymorphic bands using AFLP primer; Massawe et al.^14^ recorded the highest 88.2% polymorphism using RAPD primers, Fatimah and Ardiarini^60^, noted 73.10% polymorphism using RAPD in *V. subterranean.* The average gene flow (*Nm* = 1.54) was estimated by Oumer et al.^27^ using ISSR whereas in our study it was recorded as *Nm* = 2.26.

### Primer efficiency analysis

According to Amiryousefi et al.^43^, there are two major dimensions of genomic marker polymorphism excellency and informativeness as heterozygosity (H) and the polymorphic information content (PIC). These indices were measured based on data gained from ISSR primers using the *iMEC* (online marker efficiency calculator). The range of H and PIC value for a binary or dominant marker is maximum as 0 (monomorphic) to 0.5 (highly judicial, with multiple alleles in an identical frequency) due to assume of two alleles per locus and both are influenced by the number and frequency of alleles^61^. Estimation of PIC value delivers a projection of discriminatory power of a locus by considering not only alleles numbers but also the relative frequencies of those alleles^62^. Polymorphic information content (PIC) is the likelihood of exposure of marker polymorphism depending on the number of detectable alleles and their frequency distribution. Moreover, the PIC index indicated better sources of variation that will assist plant breeders to assess genetic diversity and inter or intra relationships among genotypes. Resolving power (RP) is an index of the separating ability of a certain marker and an effective multiplex ratio (EMR) is a matrix which highly depends on the polymorphic extent of markers. Considering the range High PIC and H values indicate the advanced discriminatory capacity of both marker systems. MI highlights the distinctive power of the primer. A higher value of Discriminating (D) power (closer to 1) indicates a lower possibility of a mix-up between *V. subterranea* accessions^43^. There was a positive correlation was observed among PIC, RP, MI, and EMR which is supported by Kayis et al.^63^ and Ramzan et al.^64^. Most of the ISSR primers were highly polymorphic and informative, suggested for genetic discrimination analysis of this studied genus. The mean of primer efficiency index was comparatively high (RP = 5.30, MI 0.675, D = 0.96) and this matrix indicates the overall efficacy of the tested primers which provides exact differentiation among the accessions. The greater the RP and MI indices refer to the greater efficacy of the respective primer^65^. In our research, the primer ISSR 842 (MI = 1.37, RP = 4.77); UBC 836 (MI = 1.28, RP = 9.27); ISSR 848 (MI = 1.14, RP = 7.22); ISSR 811 (MI = 0.99, Rp = 6.18); and ISSR 11 (MI = 0.91, RP = 7.09) had moderate to high values of marker index and resolving power, suggesting these primers are extremely potential and useful for genetic discrimination of the *V. subterranea* accessions. These findings have harmony with the previous result reported by Oumer et al.^27^; Zarei and Erfani-Moghadam et al.^65^; Ahmed et al.^61^.

### Genetic distance (Nei’s measure) analysis

In our study, besides the closely associated and distant genotypes, we recorded merely similar genetic distance values with minute fractions within the populations that reflected the extent of variation presences among the *V. subterranea* accessions evaluated. On behalf of these findings, there was clear evidence by the previous observation by Mohammed et al.^3^ and Rungnoi et al.^1^ in *V. subterranea*. In addition to detecting the genetic distances with minute, numeric fraction values emphasize the efficiency of the ISSR primers to differentiate among the Bambara groundnut genotypes, even those that have a close relationship with each other. The findings of the current study revealed the significant genetic distinction among the *V. subterranea* genotypes of GD = 0.14 to 0.39 which is consistent with the similar trends of findings reported by Massawe et al.^14^ from GD = 0.55 to 0.92 using RAPD, Siise and Massawe^20^, from 0.48 to 0.90, Mohammed et al.^3^ from GD = 0.00 to 3.8 and Somta et al.^19^ from GD = 0.27 to 0.53 using SSR markers in *V. subterranea*. Alghamdi et al.^66^ revealed considerable variation among 34 Faba bean genotypes extended from 0.22 to 0.92. The genetic distance spanned from 0.08 to 1.17 among 105 Bambara groundnut genotypes noted by Odongo et al.^22^ using microsatellite markers, whereas Ntundu et al.^17^ reported genetic distance varied from GD = 0.10 to 0.68 for 100 Bambara genotypes using AFLP markers. There was high similarity covered from 0.83 to 0.94 recorded for 12 Bambara groundnut genotypes using RAPD by Fatimah and Ardiarini^60^. The accessions with high relatedness from two different geographical zones suggesting the involved genotypes may have the common origin and/or mechanical mixture of seeds from one agro-ecological zone to another across Nigeria. The genotypes with less relatedness indicating the presence of extreme divergence among the evaluated accessions. Typically, the genetic diversity of a population in a species is influenced by several evolutionary factors, such as geographic distance, natural selection, reproductive system, gene flow, seed dispersal as well as the center of diversity^67^. However, a significant extent of genetic diversity is predictable in *V. subterranean* accessions due to geographic dispersal of the genus but differentiation among the collected germplasm is inadequate at growing areas due to mixing of germplasms within the regions and the fact is that farmers either produce their seeds or collected seeds from unauthorized ways. As a result, the existence of close relatedness was noted among some of the accessions used in this study due to the accessions collected from similar locations or origins or names of different landraces.

### Genetic relationship

Based on the ISSR banding profile we discovered three major clusters with six subclusters of the accessions evaluated in this study. The findings in our study illustrated the efficacy of ISSR markers partitions the accessions into closely related genetic groups than another marker system. The major cluster I and II occupied the maximum number of accessions whereas the smallest cluster was major cluster III which had only three genotypes from different origins. The accessions that positioned the same cluster with different regions of collection, reflecting a close genetic association despite their diverse origins. Our finding was validated by Mohammed et al.^3^ stated the seven clusters of 50 Bambara groundnut species and Odongo et al.^22^ reported three clusters of 105 Bambara groundnut genotypes using SSR primers. Conversely, the genomic grouping of Bambara groundnut accessions related to geographical distribution based on RAPDs and AFLP reported by Amadou et al.^13^ and Ntundu et al.^17^, respectively. Generally, the genotypes were positioned more closely in our generated phylogenetic tree suggesting that they were genetically more similar having identical genes. On the other hand, accessions that possess the distant group suggesting that they were genetically dissimilar even though they come from the same population as well as similar origins. This circumstance may prompt by some factors such as the mixture of seeds, mating system, natural selection, spontaneous mutation, additionally the local farmer produces their seeds or purchase from neighboring markets. A similar trend of the result was proposed in their study of Bambara groundnut by Massawe et al.^14^ using RAPD, Somta et al.^19^ using SSR, Mukakalisa et al.^15^ using RAPD, Olukolu et al.^5^ using DArT and Rungnoi et al.^1^ using RAPD, ISSR markers. Fatimah and Ardiarini^60^, grouped 12 accessions of *V. subterranea* into two clusters based on similarity indices using RAPD markers.

### Principal component analysis (PCA)

The result of PCA is generally explained in terms of component scores and loadings^68^. The principal component analysis is a method of data reduction where correlated variables are grouped and separated from others with low or no correlation. The principal component analysis is a method to explore and to visualize similarities or dissimilarities of data and assigns for each sample place in a low-dimensional space, e.g., as a 2D or 3D graphic. PCA attempts to discover the principal axes through a matrix of eigenanalysis and eigenvectors. Eigenvalues are usually ranked from the greatest to the least. The first eigenvalue is often called the "dominant" or "leading" eigenvalue. Eigenvalues are also often called "latent values". The values recorded in principal axes of PCA arebeing advocated in the similar trends of result noted by Arolu et al.^69^. In our study, the PCA analysis showed maximum variation captured by PC1 (13.92%) and PC2 (12.59%) which is supported by Rungnoi et al.^1^ and stated 90.3% variation led by the first three PCs using ISSR and RAPD in PCoA analysis of *V. subterranea*; Odongo et al.^22^ zanalyzed PCoA and concluded that 84.30% of total variability spanned by the first three PCs using SSR primer in *V. subterranea* which is higher than our findings; Molosiwa et al.^21^ noted 37.3% (1st two PCs) and 19.5% (1^st^ two PCs) of the total variation for DArT and SSR, respectively for PCoA analysis in *V. subterranea*. Kaur et al.^30^ accounted 81.13% (1^st^ 10 PCs), 61.75% (1^st^ 5 PCs), and 46.17% (1^st^ 3 PCs) variation in 23 V*. radiata* genotypes using RAPD, ISSR and SSR primers. Based on the standard Shannon diversity index (range from 1.5 to 3.5) noted by Khan et al.^70^, our calculated values indicate the presence of a moderate to high extent of genetic diversity among the accessions.

### Admixture analysis

The population structure of Bambara groundnut individuals assessed by Bayesian admixture analysis indicated 3 clusters consistent with four agro-ecological regions. Our findings of STRUCTURE analysis were in a similar trend with the genotypic relatedness revealed by UPMGA clustering resulted in entire accessions into three distinct clusters. Out of 44 accessions, 35 were comparatively pure according to Q > 0.60^49^ as the purity standard other 9 accessions were highly complex, indicating these accessions were genetically admixture. The real fact of this mixture is either more introduction of accessions from different origins or amalgamation into breeding or natural selection which leads to increased heterozygosity. The current finding is consistent with the similar trend of results reported by Rungnoi et al.^1^ estimated ΔK = 2 using ISSR and RAPD in 363 Bambara groundnut genotypes while Olukolu et al.^5^ reported ΔK = 4 using DArT assay of 40 Bambara groundnut genotypes. Additionally, other researchers had the parallel statement such as Wu et al.^49^ found ΔK = 3 using ISSR; Nilkanta et al.^42^ found ΔK = 3 using ISSR; Zarei and Erfani-Moghadam et al.^65^ found ΔK = 3 using SCoT; Barbosa et al.^71^ found ΔK = 3 using ISSR; Zimisuhara et al.^51^ found ΔK = 2 using ISSR; Li and Zhang^72^ found ΔK = 2 using ISSR.

### Fixation index (F*st*) analysis

The structure analysis further leads to the sharp emergence of three genetic groups of *V. subterranea* and the phenomenon of genetic drift or gene flow among the accessions was detected to some extent. Fixation index (F*st*) estimation helps to know how different a group of populations from each other. High F*st* implies a considerable degree of differentiation among populations. F*st* values can range from 0 to 1, where 0 means complete sharing of genetic material (two population can interbreeding freely) or panmictic population and 1 means all genetic variation is explained by the population structure, and that the two populations do not share any genetic diversity, or the populations are fixed^73^. A standard scale of fixation index is F*st* < 0.05 = little genetic difference; F*st* = 0.05–0.15 = moderate genetic difference; F*st* = 0.15–0.25 = great genetic difference; F*st* > 0.25 = very great genetic difference established by Hartl and Clark,^71^. Moreover, F*st* > 0.15 = significant differentiation and F*st* < 0.05 = insignificant differentiation reported by Frankham et al.^74^. Our estimated F*st* was 0.1896, 0.3684, and 0.3997 for cluster 1, cluster 2, and cluster 3, respectively. Considering the above scale, suggested that the population under cluster 1 showed great genetic differentiation whereas the population under cluster 2 and 3 showed very great genetic diversity. Frequent gene flow led to a low level of genetic differentiation with a small genetic distance among them. Oppositely, low gene flow governs the plant's adaptation to different growing regions influencing the higher level of genetic differentiation with greater genetic distance. Genetic enhancement of crops depends on the extent of genetic differentiation among accessions. The currently estimated fixation index using ISSR is higher than the report published by Kumar et al.^75^ F*st* = 0.17, and Kimaro et al.^76^ while lower as compared to F*st* = 0.94 reported by Kassa et al.^77^.

## Conclusion

This is the forerunner initiative on the valuation of genetic differentiation and population structure of *V. subterranea* genotypes using ISSR primers in Malaysia. Genetic relatedness and population structure are crucial for plant breeding schemes for this crop improvement as well as its conservation. Considering this intent, to conduct this study, ISSR primer was used and exhibited a moderate to high level of efficiency in assessing genetic differentiation and genetic structure in *V. subterranea* populations. The amplification of many polymorphic loci indicated the used set of ISSR primers have the potential to the assessment of genetic diversity among the existing accessions. However, the combination and a large number of molecular markers (dominant and co-dominant) to further assessment of genetic variation is highly advocated. In terms of diversity indices and genetic relationships, a significant proportion of variation was accounted for among the evaluated accessions and the diverse genotypes are suggested to use in a breeding system. Oppositely, the genotypes with low average diversity indicated the potential risk of declining genetic variation due to limited genetic basis, which alarming or enlightening the implication of biodiversity, assembling, and conserving their wild genetic resources. Moreover, the Structure, PCA, UPMGA, and Nei’s analysis divulged the entire accessions into three distinct genetic components based on ISSR amplified genomic data sets. Furthermore, fixation index (F*st*) and genetic structure with admixture analysis revealed the persistent genetic drift among the gene pool of *V. subterranea* accessions. Typically, this investigation provides an initial scientific basis of genetic data for this crop enhancement and conservation policies in the future. The result of this study will assist in more accurate portrayal, classification, preservation, and maximum utilization of genetic resources and may have real implications in future breeding schemes to broaden the genetic diversity of *V. subterranea* species.

## Supplementary Information

Supplementary Information.

## Data Availability

All data are available in the text body of the manuscript. We also confirm that, a voucher specimen of the identified species has been deposited in a publicly available herbarium and GenBank, ITAFoS, Universiti Putra Malaysia (UPM). The deposition number- Bambara groundnut (*Vigna subterranea*) /ITAFoS/UPM/S4-2020.
