# Author Correction: DNA fingerprinting, fixation-index (Fst), and admixture mapping of selected Bambara groundnut (*Vigna subterranea* [L.] Verdc.) accessions using ISSR markers system

**DOI:** 10.1038/s41598-021-96946-9

**Published:** 2021-08-26

**Authors:** Md Mahmudul Hasan Khan, Mohd Y. Rafii, Shairul Izan Ramlee, Mashitah Jusoh, Md Al Mamun, Jamilu Halidu

**Affiliations:** 1 Laboratory of Climate-Smart Food Crop Production, Institute of Tropical Agriculture and Food Security (ITAFoS), Universiti Putra Malaysia (UPM), UPM Serdang, 43400 Selangor, Malaysia; 2 Department of Crop Science, Faculty of Agriculture, Universiti Putra Malaysia (UPM), UPM Serdang, 43400 Selangor, Malaysia; 3 Bangladesh Agricultural Research Institute (BARI), Gazipur, 1701 Bangladesh; 4 Bangladesh Jute Research Institute (BJRI), Dhaka, Bangladesh

Correction to: *Scientific Reports* 10.1038/s41598-021-93867-5, published online 15 July 2021

In the original version of this Article Mohd Y. Rafii was incorrectly affiliated with ‘Bangladesh Agricultural Research Institute (BARI), Gazipur, 1701, Bangladesh’. The correct affiliations are listed below.

Laboratory of Climate-Smart Food Crop Production, Institute of Tropical Agriculture and Food Security (ITAFoS), Universiti Putra Malaysia (UPM), UPM Serdang, 43400, Selangor, Malaysia.

Department of Crop Science, Faculty of Agriculture, Universiti Putra Malaysia (UPM), UPM Serdang, 43400, Selangor, Malaysia.

The original Article has been corrected.

