# Supplementary material for: DNA fingerprinting, fixation-index (Fst), and admixture mapping of selected Bambara groundnut (Vigna subterranea [L.] Verdc.) accessions using ISSR markers system
Source: Sci Rep. 2021 Jul 15;11:14527. doi: 10.1038/s41598-021-93867-5 (PMC8282841; doi:10.1038/s41598-021-93867-5)
Supplement: Supplementary file 1 — Supplementary Information. [file 41598_2021_93867_MOESM1_ESM.doc]

**Supplementary Information**

**DNA Fingerprinting, Fixation-index (Fst) and Admixture mapping of Selected Bambara groundnut (*Vigna subterranea* [L.] Verdc.) Accessions using ISSR Markers System**

Md Mahmudul Hasan Khan^1, 3*^, Mohd Y. Rafii^1, 2*^, Shairul Izan Ramlee^2^, Mashitah Jusoh^2^, Md Al Mamun^1, 4^, and Jamilu Halidu^1^

^1^Laboratory of Climate-Smart Food Crop Production, Institute of Tropical Agriculture and Food Security (ITAFoS), Universiti Putra Malaysia (UPM), 43400, UPM Serdang, Selangor, Malaysia.

^2^Department of Crop Science, Faculty of Agriculture, Universiti Putra Malaysia (UPM), 43400, UPM Serdang, Selangor, Malaysia.

^3^ Bangladesh Agricultural Research Institute (BARI), Gazipur-1701, Bangladesh.

^4^Bangladesh Jute Research Institute (BJRI), Dhaka, Bangladesh.

*Correspondence: M.Y. Rafii; mrafii@upm.edu.my, and M.M.H. Khan; [mhasan.bari12@gmail.com](mailto:mhasan.bari12@gmail.com). Tel.: +60-3-97691043,

**Supplementary tables**

Table S1: Selected thirty-two ISSR primers used in this study and their properties (sequences, base pair, GC content, melting, and annealing temperature

| **Markers** | **Sequences** | **GC%** | **DNA Bases** | **Tm** | **Ta** | **References** |
| --- | --- | --- | --- | --- | --- | --- |
| ISSR 11 | AGCAGCAGCAGCAGC | 66.7 | 15 | 56.7 | 59.7 | [1] |
| ISSR 18 | CACACACACACACACA | 50 | 16 | 49.6 | 58.6 | [1] |
| UBC 807 | AGAGAGAGAGAGAGAGT | 47.1 | 17 | 47 | 48.1 | [29] |
| UBC 808 | AGAGAGAGAGAGAGAGC | 52.9 | 17 | 48.8 | 49.6 | [29] |
| UBC 809 | AGAGAGAGAGAGAGAGG | 52.9 | 17 | 48.2 | 51.6 | [29] |
| UBC 810 | GAGAGAGAGAGAGAGAT | 47.1 | 17 | 45.4 | 46.3 | [29] |
| UBC 816 | CACACACACACACACAT | 47.1 | 17 | 50.1 | 50.8 | [29] |
| UBC 836 | AGAGAGAGAGAGAGAGYA | 47.2 | 18 | 48.9 | 47.3 | [29] |
| UBC 841 | GAGAGAGAGAGAGAGAYC | 52.8 | 18 | 48.5 | 49.8 | [29] |
| UBC 844 | CTCTCTCTCTCTCTCTRC | 52.8 | 18 | 48.6 | 49.8 | [29] |
| UBC-815 | CTCTCTCTCTCTCTCTG | 52.9 | 17 | 46.8 | 51.4 | [30] |
| UBC 817 | CACACACACACACACAA | 47.1 | 17 | 50.3 | 51.5 | [30] |
| UBC 873 | GACA GACA GACA GACA | 50 | 16 | 47.4 | 42.4 | [30] |
| ISSR 811 | ACACACACACACACT | 46.7 | 15 | 47.3 | 48.3 | [31] |
| ISSR 901 | AGAGAGAGAGAGAGAGYC | 52.8 | 18 | 50.2 | 49.1 | [31] |
| UBC 835* | CTCTCTCTCTCTCTCAT | 47.1 | 17 | 45.7 | 44.9 | [31] |
| ISSR 889 | GAGAGAGAGAGAGAGATT | 44.4 | 18 | 46.3 | 42.6 | [31] |
| ISSR 812 | GAGAGAGAGAGAGAGAA | 47.1 | 17 | 45.7 | 41.4 | [32] |
| ISSR 842 | GAGAGAGAGAGAGAGACTG | 52.6 | 19 | 50.2 | 51.5 | [32] |
| A-856 | ACACACACACACACACYA | 47.2 | 18 | 52.8 | 57.8 | [33] |
| I-825 | ACACACACACACACACAT | 44.4 | 18 | 52 | 57.8 | [34] |
| ISSR 10 | AGA GAG AGA GAG AGA GYC | 52.8 | 18 | 50.2 | 47.4 | [35] |
| ISSR 17 | TCT CTC TCT CTC TCT CRG | 52.8 | 18 | 50 | 49.1 | [35] |
| PRIMER 9 | AGAGAGAGAGAGAGAGAGAGT | 47.6 | 21 | 52.8 | 47 | [36] |
| ISSR 856 | ACCATGGCTACCACCGAC | 61.1 | 18 | 57.3 | 52.3 | [31] |
| ISSR 2M | CACACACACACACACAAAGCT | 47.6 | 21 | 56.3 | 61.3 | [32] |
| UBC 835** | AGAGAGAGAGAGAGAGYC | 52.8 | 18 | 50.2 | 41..4 | [29] |
| UBC 813 | CTCTCTCTCTCTCTCTT | 47.1 | 17 | 45.7 | 41.7 | [30] |
| PRIMER 3 | CTCCTCCTCCTCCTCCTC | 66.7 | 18 | 55.7 | 54.2 | [36] |
| ISSR 848 | CACACACACACACACAAAGG | 50 | 20 | 54.5 | 57.4 | [32] |
| UBC 825 | ACACACACACACACACT | 47.1 | 17 | 51.4 | 53.1 | [34] |
| UBC 830 | TGTGTGTGTGTGTGTGG | 52.9 | 17 | 52.7 | 56 | [29] |

Legend: R = A, G (Purine); Y = C, T (Pyrimidine); **Tm =** melting temperature; **Ta =** annealing temperature; * and ** Both (UBC 835) name are same as per source of collection, but the sequences are not same and collected from two different sources

**Supplementary Figure**

Figure S1:

**Full Gels/blots picture**


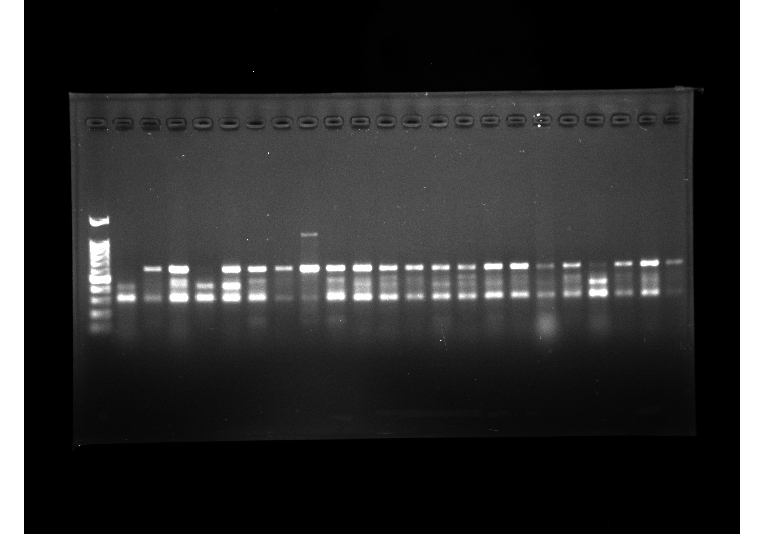


UBC 873

**(a). UBC 873 (Genotype: G1 to G22)**

M G1 G2 G3 G4 G5 G6 G7 G8 G9 G10 G11 G12 G13 G14 G15 G16 G17 G18 G19 G20 G21 G22

1100-

700-

200-

bp


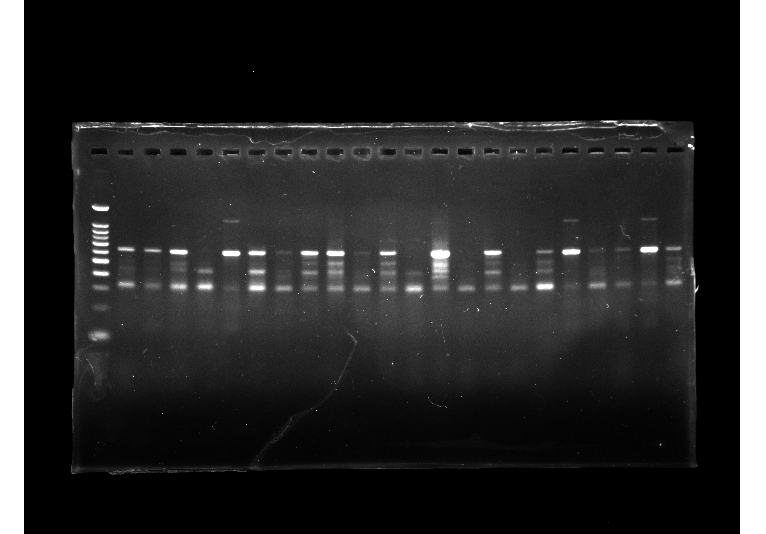


UBC 873

**(a). UBC 873 (Genotype: G23 to G44)**

M G23 G24 G25 G26 G27 G28 G29 G30 G31 G132 G33 G34 G35 G36 G37 G38 G39 G40 G41 G42 G43 G44

200-

700-

1100-

bp


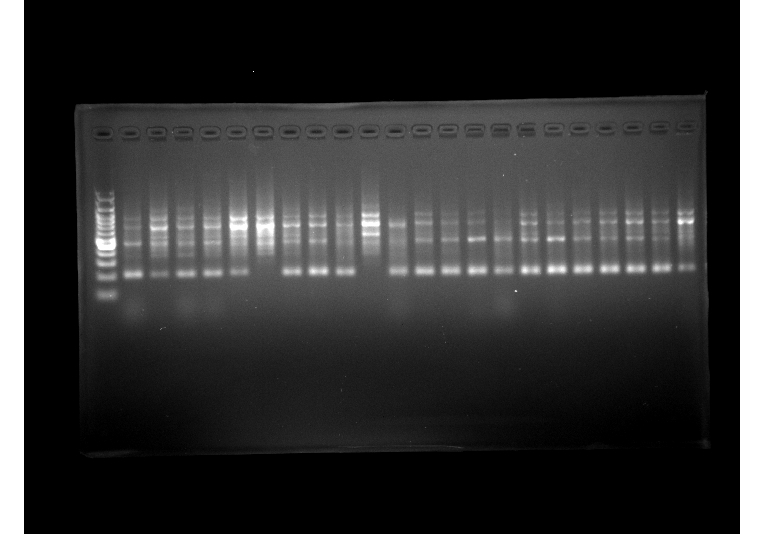


ISSR 11

bp

100-

700-

1100-

**(b). ISSR 11 (Genotype: G1 to G22)**

M G1 G2 G3 G4 G5 G6 G7 G8 G9 G10 G11 G12 G13 G14 G15 G16 G17 G18 G19 G20 G21 G22


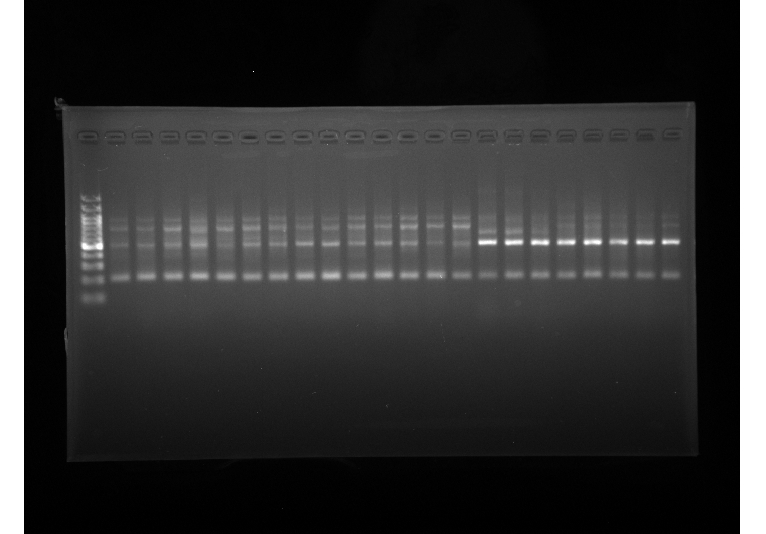


bp

500-

1100-

**(b). ISSR 11 (Genotype: G23 to G44)**

ISSR 11

M G23 G24 G25 G26 G27 G28 G29 G30 G31 G132 G33 G34 G35 G36 G37 G38 G39 G40 G41 G42 G43 G44

200-

ISSR 11


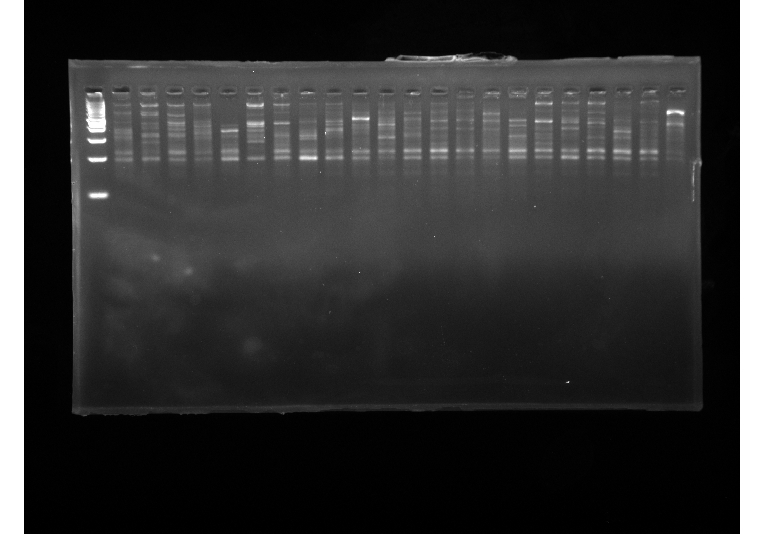


bp

200-

300-

600-

**(c). UBC 807 (Genotype: G1 to G22)**

UBC 807

M G1 G2 G3 G4 G5 G6 G7 G8 G9 G10 G11 G12 G13 G14 G15 G16 G17 G18 G19 G20 G21 G22

UBC 807


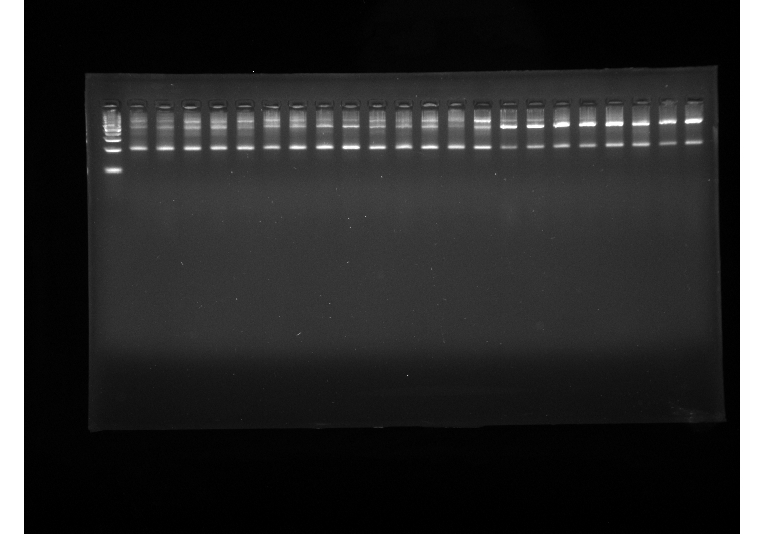


UBC 807

bp

100-

500-

700-

**(c). UBC 807 (Genotype: G23 to G44)**

M G23 G24 G25 G26 G27 G28 G29 G30 G31 G132 G33 G34 G35 G36 G37 G38 G39 G40 G41 G42 G43 G44

Figure S1: Inter Simple Sequence (ISSR) banding profiles of 44 *V. subterranea* accessions. The full-length blots/gels of a) the UBC 873 primer; b) ISSR 11 primer, and c) UBC 807 amplified with Agarose gel electrophoresis using IMAGELAB ver. 5.0 program (BIORAD). In each gel we run 22 accessions at a time and lane M refers to 100 bp DNA ladder and lane with numeric number refers to the accession number listed in Table 1.
